# *In silico*, *in vitro*, and *in vivo* characterization of thiamin-binding proteins from plant seeds

**DOI:** 10.1042/BCJ20240429

**Published:** 2025-02-05

**Authors:** Maria Faustino, Simon Strobbe, Raul Sanchez-Muñoz, Da Cao, Ratnesh C. Mishra, Tiago Lourenço, M. Margarida Oliveira, Dominique Van Der Straeten

**Affiliations:** 1 Laboratory of Functional Plant Biology, Ghent University, K. L. Ledeganckstraat 35, B-9000 Gent, Belgium; 2 Laboratory of Plant Functional Genomics, Instituto de Tecnologia Química e Biológica António Xavier, Universidade Nova de Lisboa, 2780-157, Oeiras, Portugal

**Keywords:** molecular docking, seed storage protein, thermal-shift assay, thiamin, vitamin B1

## Abstract

Thiamin, an essential micronutrient, is a cofactor for enzymes involved in the central carbon metabolism and amino acid pathways. Despite efforts to enhance thiamin content in rice by incorporating thiamin biosynthetic genes, increasing thiamin content in the endosperm remains challenging, possibly due to a lack of thiamin stability and/or a local sink. The introduction of storage proteins has been successful in several biofortification strategies, and similar efforts targeting thiamin have been performed, leading to a 3–4-fold increase in white rice. However, only one thiamin-binding protein (TBP) sequence has been described in plants, more specifically from sesame seeds. Therefore, we aimed to identify and characterize TBPs, as well as to evaluate the effect of their expression on thiamin concentration, using a comprehensive approach integrating *in silico*, *in vitro,* and *in vivo* methods. We identified the sequences of putative TBPs from *Oryza sativa* (Os, rice), *Fagopyrum esculentum* (Fe, buckwheat), and *Zea mays* (Zm, maize) and pinpointed the thiamin-binding pockets through molecular docking. FeTBP and OsTBP contained one pocket with binding affinities similar to the *Escherichia coli* TBP, a well-characterized TBP, supporting their function as TBPs. *In vivo* expression studies of TBPs in tobacco leaves and rice callus resulted in increased thiamin levels, with FeTBP and OsTBP showing the most pronounced effects. Additionally, thermal shift assays confirmed the thiamin-binding capabilities of FeTBP and OsTBP, as observed by the significant increases in melting temperatures upon thiamin binding, indicating protein stabilization. These findings offer new insights into the diversity and function of plant TBPs and highlight the potential of FeTBP and OsTBP to modulate thiamin levels in crop plants.

## Introduction

**Figure 1:**
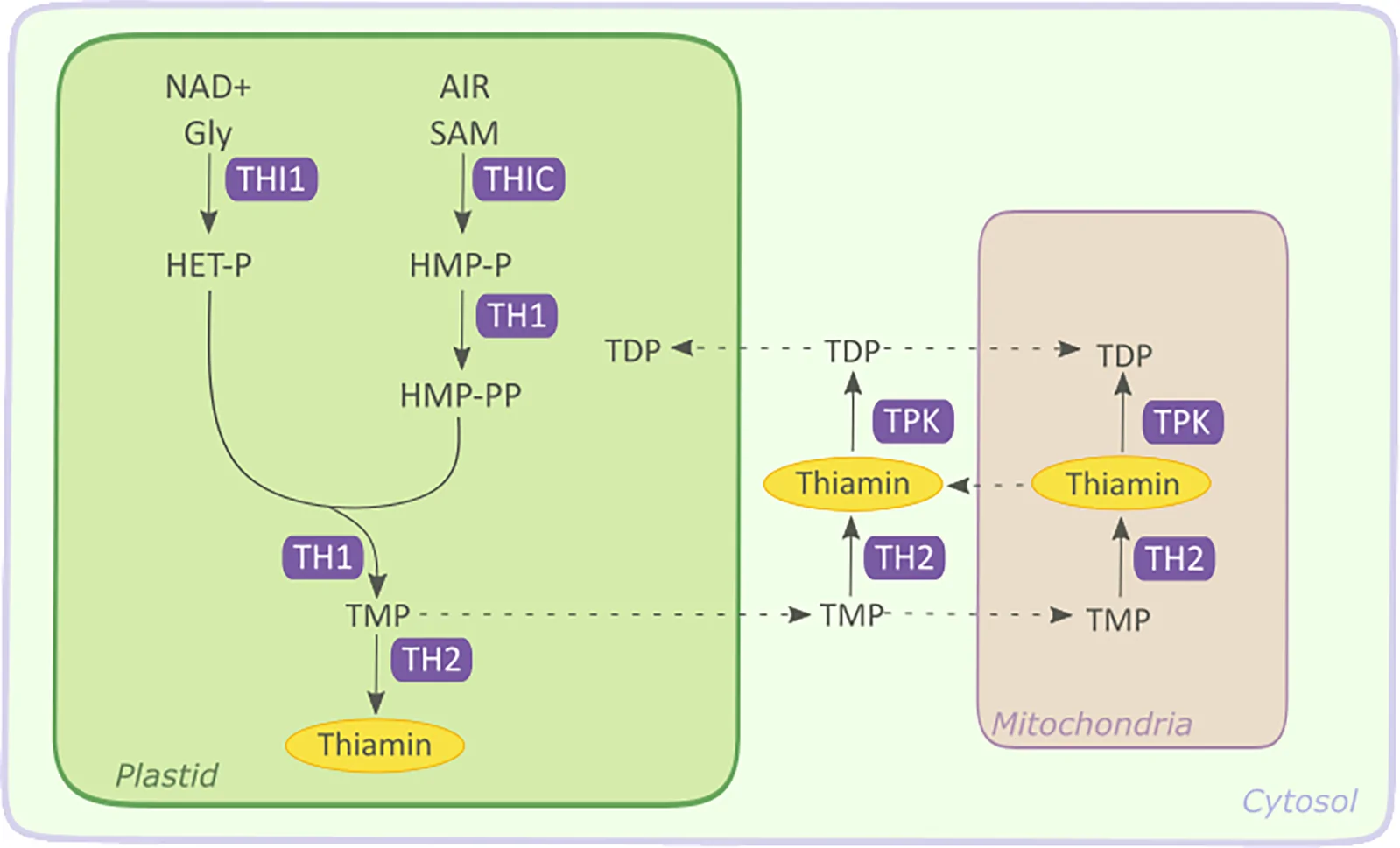
Thiamin biosynthesis in plants. Figure adapted from Strobbe and Van Der Straeten [11]. Biosynthetic pathway is depicted in grey, while enzymes are shown in purple background. Dashed arrows represent putative transport between cell compartments. The synthesis of the pyrimidine moiety (HMP-P) requires 5-aminoimidazole ribonucleotide (AIR) and S-adenosylmethionine (SAM) as substrates and is catalyzed by 4-amino-2-methyl-5-hydroxymethylpyrimidine phosphate (HMP-P) synthase (THIC) [2]. THIC mRNA precursor is regulated by a riboswitch in its 3′ untranslated region (UTR). This riboswitch functions as a TDP sensor controlling THIC mRNA stability and, consequently, TDP production [12–14]. The synthesis of the thiazole moiety (HET-P) is mediated by 4-methyl-5-β-hydroxyethylthiazole phosphate (HET-P) synthase (THI1) [4], the substrates of this reaction are nicotinamide adenine dinucleotide (NAD) and glycine [4]. TH1 (HMP-P kinase/thiamin monophosphate (TMP) pyrophosphorylase) phosphorylates HMP-P to HMP-PP and also condenses HMP-PP and HET-P to form TMP [7]. TH2 dephosphorylates TMP [8,9,15] which is then pyrophosphorylated to TDP by TDP kinases (TDPKs) [10].

Thiamin, particularly thiamin diphosphate (TDP), serves as a cofactor for several enzymes of the central carbon metabolism [1]. These enzymes are integral to tricarboxylic acids and other acetyl Co-A-dependent pathways (e.g. fatty acids synthesis/breakdown and mevalonate-dependent pathways), as well as to amino acid pathways [1]. The thiamin biosynthetic pathway comprises two separate branches that lead to the synthesis of a pyrimidine (HMP-P) [2,3] and a thiazole ring moiety (HET-P) [4,5]. These are condensed to form thiamin monophosphate (TMP) [6,7], which is subsequently converted into thiamin [8,9] and into the active form, TDP [10] (Figure 1). Thiamin metabolism is highly complex, and much remains to be discovered regarding salvage mechanisms, regulatory processes, and storage [16].

From a nutritional point of view, the engineering of storage proteins in plant seeds has gathered attention since it can lead to increased vitamin stability and/or content. For instance, the long-term stabilization of folate (vit.B9) in rice was obtained by introducing genes encoding folate-binding proteins (up to 150-fold of wild-type rice levels when combined with folate biosynthesis genes) [17]. Exploration of *Sesamum indicum* TBPs in a similar context has been performed, leading to 3–4-fold thiamin increase in polished rice [18].

The majority of thiamin in plant seeds is found to be bound to TBPs [19]. TBPs have been identified in various plant species seeds [20,21], each exhibiting distinct molecular mass, subunit structures, amino acid composition, optimum pH for thiamin-binding activity, and affinity for thiamin analogs [22]. Despite their differences, TBPs from plant seeds are generally globulins [21].

Rice seeds harbor a globulin TBP with high thiamin specificity [23]. The presence of this rice protein decreases gradually from the outer layers to the inner layers of the grain [24], mirroring patterns observed in TBPs from wheat and sesame seeds, where TBP accumulates in the aleurone layer during seed development [22]. In this stage, thiamin-binding capacity of rice TBP increases alongside thiamin levels [25], while germination leads to a decline in activity [24]. This can be linked to proteolytic enzymes that degrade the TBP, providing an additional pool of amino acids for protein synthesis during germination [25,26]. This process causes the release of thiamin from the protein, followed by phosphorylation by thiamin pyrophosphokinase. The resulting TDP serves as a coenzyme for metabolic processes that generate the critical energy for germination [25,26]. This elegantly orchestrated mechanism serves a dual purpose: it stores amino acids and thiamin, both indispensable for the successful initiation of seed germination.

Although several studies have isolated, purified, and characterized TBPs derived from plant seeds, the gene and protein sequences remain unidentified, except for sesame seeds TBP, which belong to a different protein family [27]. In this study, we identified three TBPs from buckwheat (FeTBP), rice (OsTBP), and maize (ZmTBP) through *in silico* approaches (sequence analysis and docking assays). *In vivo* expression studies (in yeast, rice callus, and tobacco leaves) and *in vitro* assays (thermal shift assay) confirmed their putative function. By focusing on the identification and characterization of plant TBPs, we aim to contribute to the development of novel strategies for thiamin biofortification.

## Results

### *In silico* identification of *FeTBP*, *OsTBP,* and *ZmTBP*

**Figure 2:**
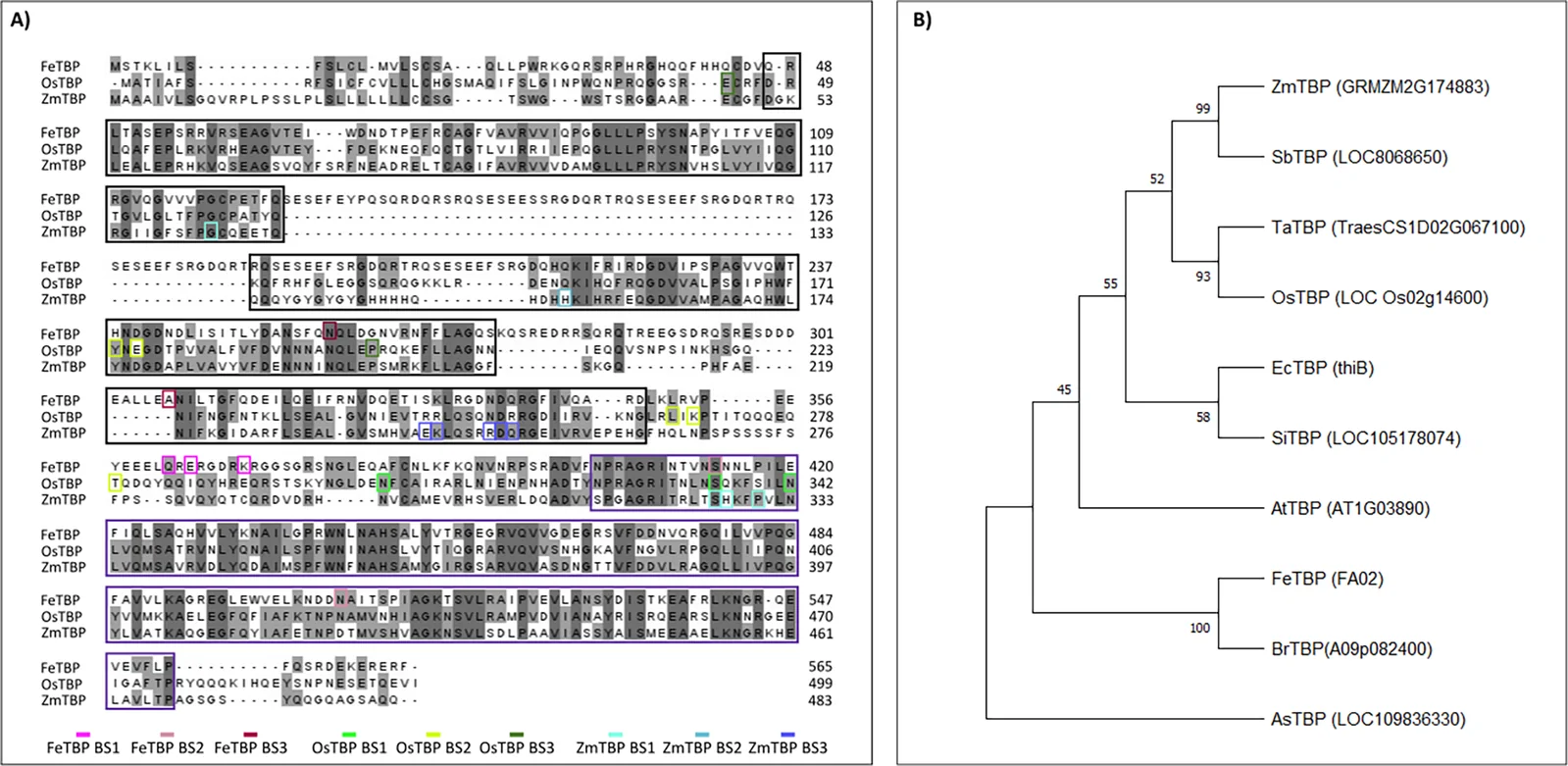
Comparative analysis of putative plant TBPs sequences and phylogenetic tree. **(A)** Alignment of putative plant TBPs. Protein sequences of *O. sativa* (OsTBP), *Z. mays* (ZmTBP), and *F. esculentum* (FeTBP). The alignment was performed using MUSCLE [30] and visualized in JalView [31]. The black and purple boxes are the conserved sequences of the N-terminal and C-terminal cupin domains, respectively. (**B**) Maximum -likelihood phylogenetic tree developed using MEGAX with the sequences of TBP proteins identified. The numbers at the branching points indicate the percentage of times each branch topology was found during bootstrap analysis (*n* = 1000). AtTBP, *A. thaliana* (AT1G03890); AsTBP, *Asparagus officinalis* (LOC109836330); BrTBP, *Brassica rapa* (A09p082400); EcTBP, *E. coli* (thiB); FeTBP, *F. esculentum* (FA02); OsTBP, *O. sativa* (LOC_Os02g14600); SiTBP, *S. indicum* (LOC105178074); SbTBP, *Sorghum bicolor* (LOC8068650); TaTBP, *Triticum aestivum* (TraesCS1D02G067100); ZmTBP, *Z. mays* (GRMZM2G174883).

Aiming to identify the sequence of TBPs from crops, we used the partial amino acid sequence of *F. esculentum* TBP [28] as a query in a BLASTp search against its complete proteome. Through this, we identified FA02 protein as the putative *F. esculentum* TBP with 96.97% identity and used it as a reference to search rice and maize total proteomes. We identified LOC_Os02g14600 as the putative rice TBP and GRMZM2G174883 as the putative maize TBP, with 37% and 34% identity to FeTBP protein, respectively. The three putative TBPs belong to the 11S seed protein family and contain a cupin 11S legumin conserved domain in the N- and C-terminals, characteristics of seed storage proteins [29] (Figure 2A).

To establish relationships among plant TBPs, we conducted a phylogenetic analysis comprising known (*E. coli* and *Sesamum indicum*) and putative TBPs (*Arabidopsis thaliana, Asparagus officinalis*, *Brassica rapa*, *F. esculentum*, *O. sativa*, *Sorghum bicolor*, *Triticum aestivum,* and *Z. mays*) (Table S1). The resulting phylogenetic tree reveals divergence among the sequences of the target putative proteins (Figure 2B). Notably, TBPs from different plants have specific molecular mass, subunit structures, amino acid composition, optimum pH for thiamin-binding activity, and affinity for thiamin analogs [22], suggesting different origins. Our phylogenetic analysis substantiates this hypothesis, highlighting the complexity of plant TBPs.

### Molecular docking analysis reveals three binding pockets for thiamin in putative *FeTBP*, *OsTBP*, and *ZmTBP*

**Figure 3:**
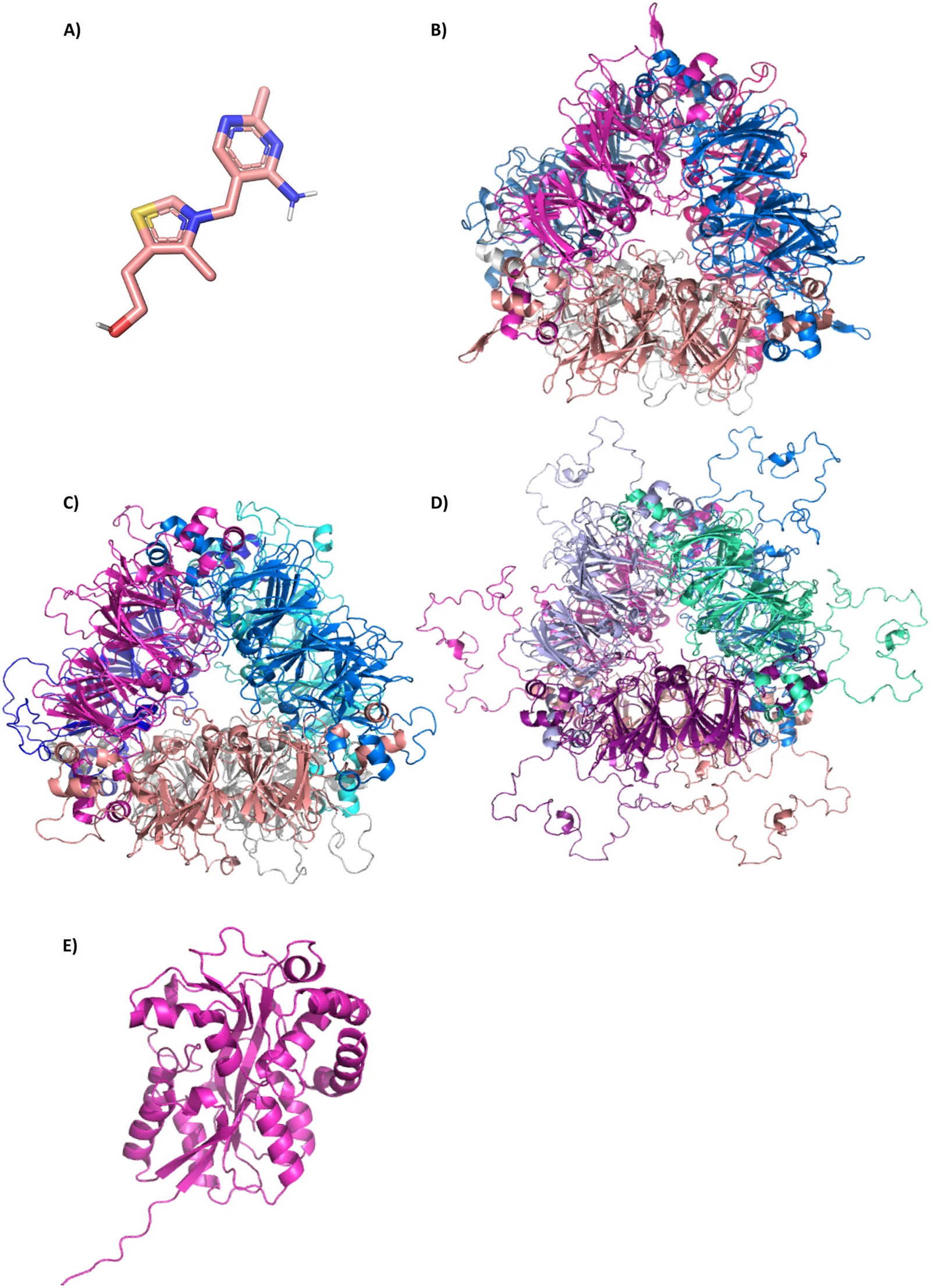
Structure of the ligand thiamin and SWISS-Model representation of the selected putative TBPs using PyMOL. (**A**) 3-D structure of thiamin. Homology models of the three putative TBPs, (**B**) ZmTBP, (**C**) OsTBP, (**D**) FeTBP, and the known **(E)** *E. coli* TBP. Plant TBPs are predicted to be hexamers (according to alpha fold predictions), while the *E.coli* one is a dimer.

Following the *in silico* identification of putative TBPs from buckwheat, rice, and maize, we performed a molecular docking analysis. From a chemical perspective, thiamin consists of 2-methyl-4-aminopyrimidine attached via a methylene group to a thiazole ring, substituted with a methyl group in the fourth position and a hydroxyethyl group in the fifth position [32]. The thiazole ring is considered the primary reaction site since the hydrogen at C2 of the ring is acidic, and therefore, the base-catalyzed ionization to an anion occurs easily [33]. Furthermore, the thiamin molecule is not considered highly flexible since it has only three basic conformations based on torsion angles [34]. We used SWISS-MODEL models of the putative proteins based on available AlphaFold GB models (Figure 3), which are reported to be more accurate than traditional homology models, with errors nearly as minor as differences between structures of the same protein determined experimentally with different ligands bound [35]. To understand the binding attributes of the three putative TBPs with the ligand thiamin, we performed a docking analysis and studied its interactions.

**Figure 4:**
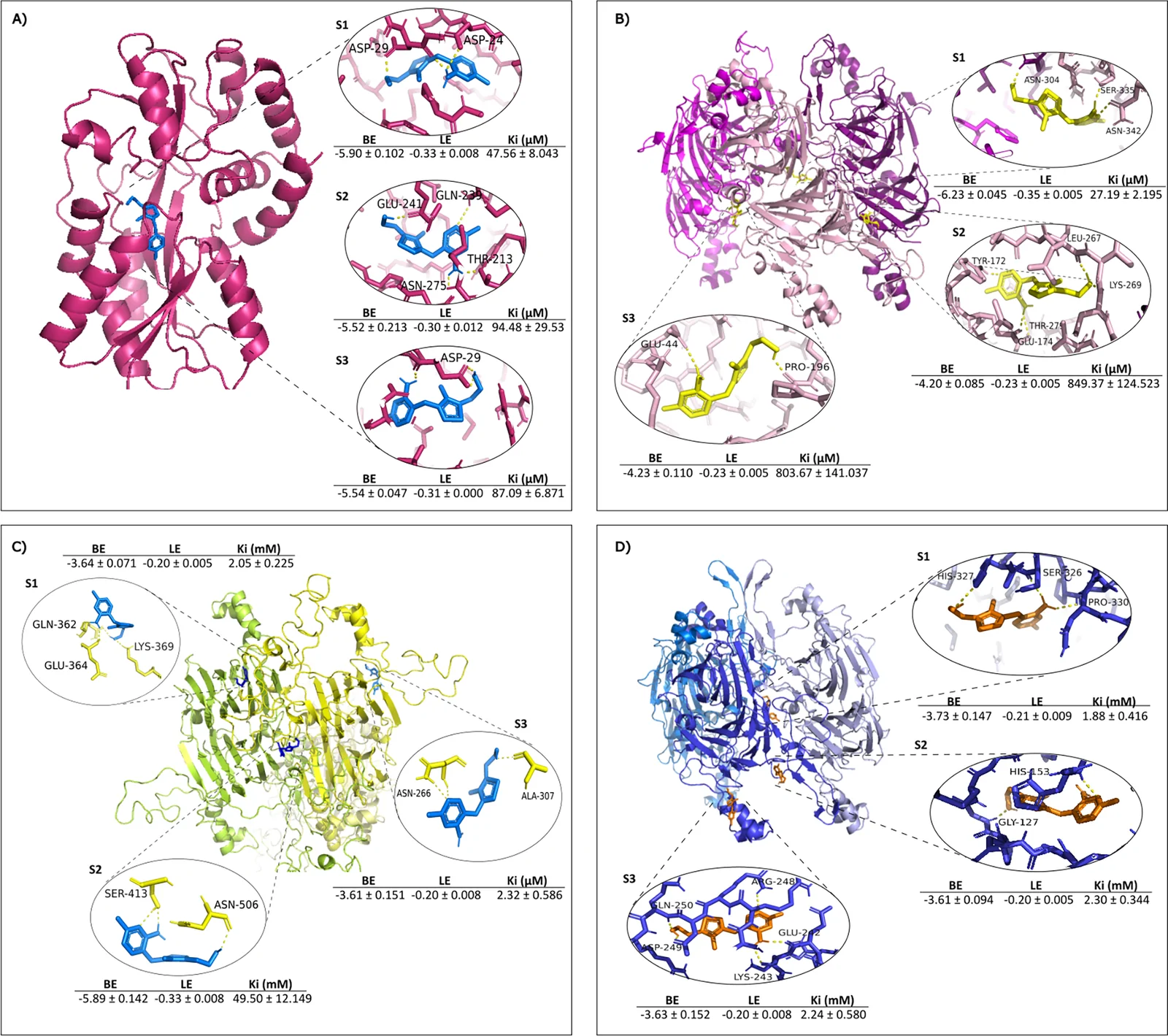
Molecular docking predictions of thiamin binding with *the putative binding proteins.* (**A**) *EcTBP;* (**B**) *OsTBP;* (**C**) *FeTBP;* (**D**) *ZmTBP*. The dotted lines represent hydrogen bonds. BE, bBinding energy; LE, ligand efficiency; Ki, iInhibitory constant. S1, S2, and S3 represent putative binding sites 1, 2, and 3, respectively.

FeTBP, OsTBP, and ZmTBP each showed three docking pockets for thiamin, contrasting with the single pocket observed in *E. coli* TBP (Figure 4). Although the model predicted three binding pockets, it was clear that, in all of them, one pocket is preferential for the binding of thiamin, which is indicated by the lower binding energy (BE) that reflects the stability of the binding complex [36] and by the lower inhibitory constant (Ki) (Figure 4). Among the putative TBPs, the BEs varied, with OsTBP S1 displaying the most favorable BE (−6.23 ± 0.045), followed by FeTBP S2 (−5.89 ± 0.142), while ZmTBP showed comparatively weaker binding (−3.73 ± 0.147) (Figure 4). Interestingly, in the preferred binding pocket across all species, thiamin interacts with a conserved serine residue through hydrogen bonds (Figure 2A). Compared with EcTBP (positive control), OsTBP S1 and FeTBP S2 pockets present similar BEs and Ki values, supporting their putative function. In OsTBP S1, thiamin interacts through hydrogen bonds with Asn-304, Ser-335, and Asn-342 (Figure 4B and Figure S1). The binding to Asn-304 occurs through a hydrogen bond between the thiamin hydroxyl group and Asn hydrogen from the amine group. It also forms hydrogen bonds with Ser-335 (hydroxyl group) and Asn-342 (carbonyl group) through thiamin hydrogens from the amine group. In the case of FeTBP S2 (Figure 4C and Figure S2), the Ser-413 hydroxyl group forms a hydrogen bond with the thiamin amine group and the neighbor nitrogen from the aromatic ring. The hydrogen from the thiamin hydroxyl group is also bound to the Asn-506 carbonyl group.

Our findings predict the structural interactions between thiamin and putative TBPs, with different species-specific binding affinities. FeTBP and OsTBP have higher affinities to thiamin, comparable to the ones observed in EcTBP, whose function has been proved. ZmTBP has lower BE and higher Ki values, which are inconsistent with a high-affinity binding between the putative TBP and thiamin. Altogether, these results support the structural interaction of thiamin with the putative TBPs, with higher affinity to FeTBP and OsTBP than ZmTBP.

### Expression of *OsTBP* and *FeTBP* results in increased thiamin content in planta

**Figure 5:**
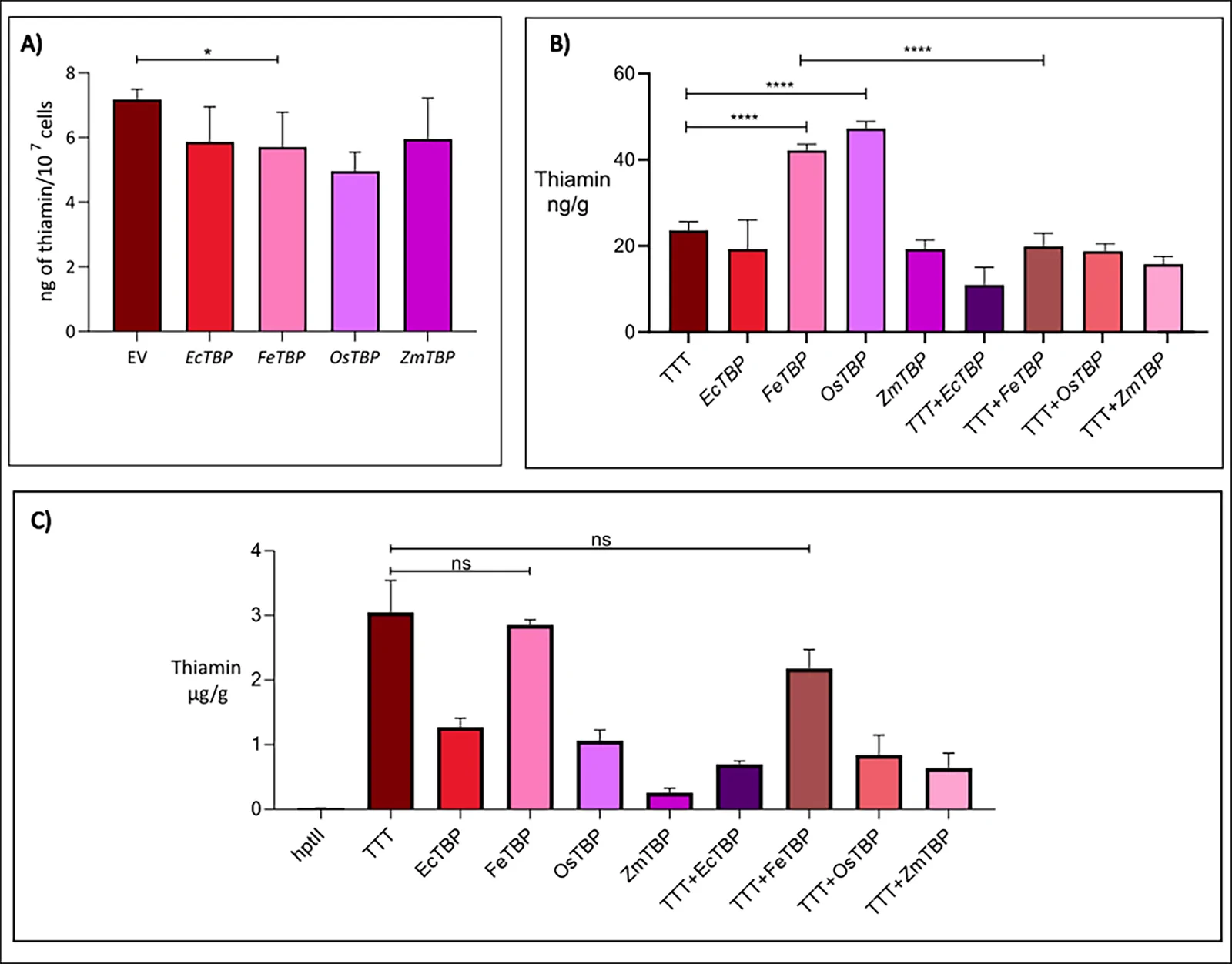
Thiamin content in yeast, tobacco leaves, and rice callus overexpressing EcTBP, ZmTBP, OsTBP, and *FeTBP*. (**A**) Thiamin content in yeast. Three independent yeast colonies were grown until OD_600_ = 1.2, washed 3 times, and stored at −80°C, *N* = 3. Data were compared with control values (EV) by One-way ANOVA (**, *P*<0.01). (**B**) Thiamin content in tobacco leaves. The area of tobacco leaves infected with Agrobacterium was collected 5 days post infiltration; the material was ground and stored at −80°C, *N* = 3. Each leaf was infiltrated with the negative control, pDGBΩ1 empty vector (left half leaf), and transgenes (right half leaf). The results represent the concentration of thiamin in the transgene half minus the one in the control half. Data were compared with positive control values (TTT) or FeTBP by One-way ANOVA (**, *P*<0.01). (**C**) Thiamin content in rice callus. Three independent lines, positive for each transgene, were selected and stored at −80°C, *N* = 3. Thiamin was detected and quantified by HPLC-MS. TTT was used as an overexpression control since this strategy has been previously employed in stable rice transformations [16]; *E. coli* TBP was used as a positive control since its function has been n [37]. Data shown correspond to means ± standard deviation of the means (SD). EV, pAG416 empty vector; TTT, combinatory expression of *OsTH1* (HMP-P kinase/thiamin monophosphate (TMP) pyrophosphorylase), *OsTHI1* (4-methyl-5-β-hydroxyethylthiazole phosphate (HET-P) synthase), and *OsTHIC* (4-amino-2-methyl-5-hydroxymethylpyrimidine phosphate (HMP-P) synthase).

To elucidate the putative binding capacities of OsTBP, FeTBP, and ZmTBP, we performed expression studies across different biological systems, including yeast, rice callus, and tobacco leaves (Figure 5). The underlying rationale was rooted in the hypothesis that if these proteins harbor thiamin-binding capabilities, their expression would trigger an increase in thiamin content, as found for folate content in folate-binding protein overexpressing transgenic rice [17]. To comprehensively evaluate the extent of their impact, we co-expressed the putative TBPs with three thiamin biosynthetic genes – *TH1*, *THI1*, and THIC (collectively referred to as TTT). The TTT approach has been previously employed in stable rice transformations for biofortification purposes [16], also serving as suitable overexpression control in our experimental design. *E. coli* TBP was used as positive control since its function is proven [37]. Tobacco leaves were harvested 4 and 5 days post infiltration and, 5 days after infiltration, were chosen to analyze thiamin content based on gene expression (Figure S1).

Upon expression of the putative TBPs in yeast (Figure 5A), no significant change in thiamin concentration was observed. Nonetheless, the transient expression of TBPs in tobacco leaves resulted in a substantial increase in thiamin concentration compared with the TTT expression alone (Figure 5B). *E. coli* TBP and maize TBP yielded a similar concentration of thiamin as the TTT. Notably, buckwheat and rice TBPs expression resulted in a 2-fold increase in thiamin levels compared with TTT. In rice callus, a similar trend was observed, with buckwheat TBP yielding the highest thiamin concentration, comparable to the overexpression of TTT. OsTBP expression resulted in a thiamin concentration similar to the one obtained by expressing EcTBP but significantly lower than the expression of TTT (Figure 5C). In the case of ZmTBP, expression yielded the lowest thiamin concentration (Figure 5C), which may be related to its lower thiamin affinity, as predicted by the docking analysis. In transient expression in tobacco leaves and upon expression in rice callus, combining TBP expression with TTT led to lower thiamin concentration than individual TTT or TBP expression, except in the case of ZmTBP where TBP expression had a lower effect on thiamin concentration compared with OsTBP and FeTBP. This concentration was maintained upon combined expression of TTT and ZmTBP, suggesting potential regulatory interactions. To evaluate whether the observed lack of thiamin production was correlated with gene expression, an analysis was conducted in rice callus. No significant correlation was observed between gene expression and thiamin concentration (Figure 5), indicating that a tight thiamin regulation at the post-translational level in rice callus could be occurring, similar to the regulatory systems observed for other vitamins such as A and B6 [38,39]. Overall, our results show that the most promising TBPs are FeTBP and OsTBP.

### Thermal shift assay confirmed thiamin binding to FeTBP and OsTBP

**Figure 6:**
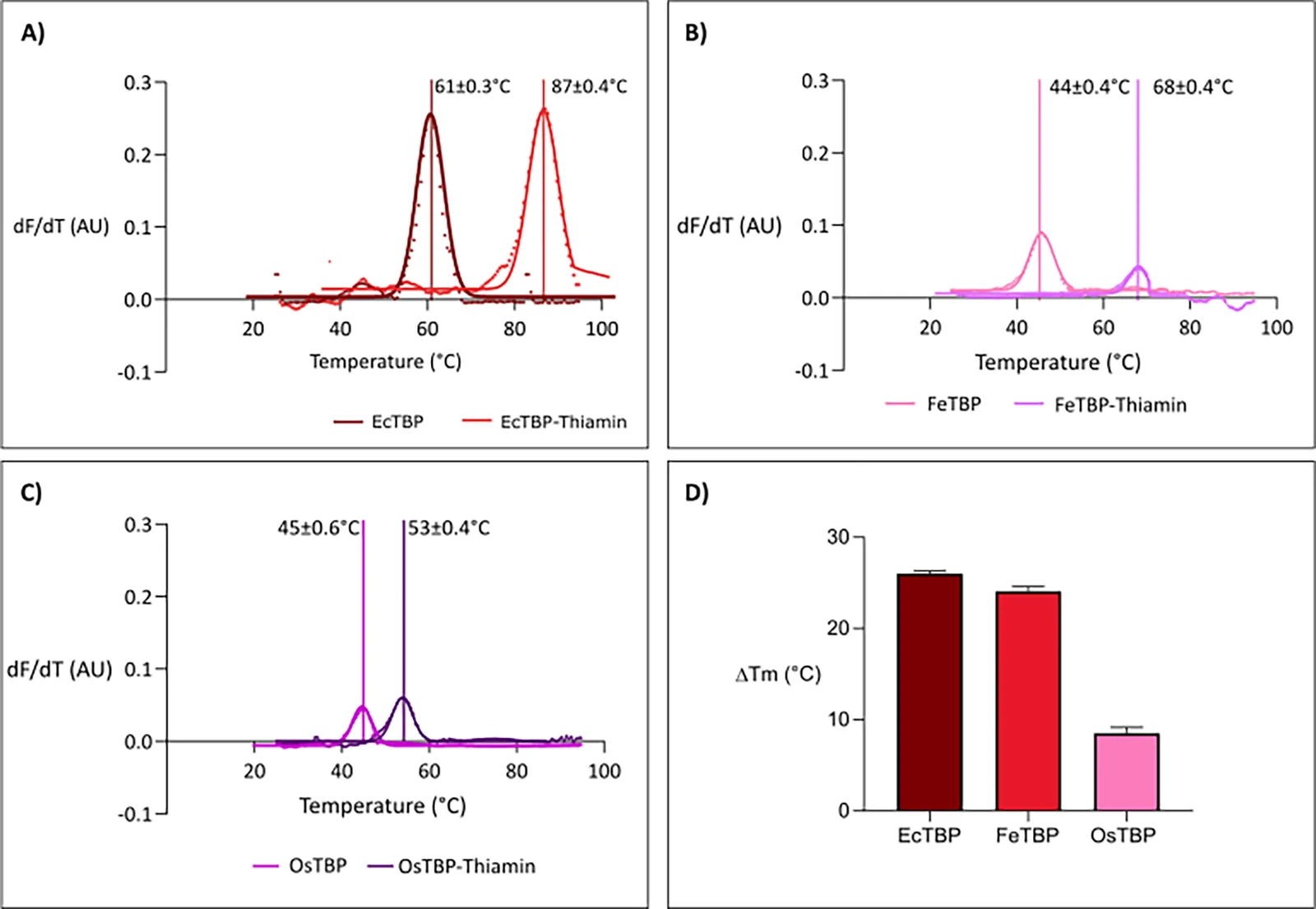
Thermal shift assay determination of thiamin-binding proteins. The melting temperatures (Tm) were selected from the first derivative of the change in fluorescence (dF/dT). Plot of the derivative of the change in fluorescence (dF/dT) for (**A**) EcTBP (5 mM), (**B**) FeTBP (3 mM), and (**C**) OsTBP (3 mM) in the presence of thiamin or buffer. (**D**) Average melting temperature difference (∆Tm) from four independent experiments for EcTBP, FeTBP, and OsTBP after adding thiamin. The respective SD errors are shown for each protein.

Since ZmTBP showed lower binding affinity to thiamin in the docking assays and poorer results in the expression studies across different plant tissues, we focused on confirming the binding of thiamin with recombinant FeTBP and OsTBP by thermal shift assay. An increase in melting temperature reflects the stability of the TBP-thiamin complex [40]. The addition of thiamin increased the thermal stability of EcTBP, FeTBP, and OsTBP by a dramatic ∆Tm of 26±0.27°C, 24.07±0.52°C, and 8.51±0.64°C, respectively (Figure 6). FeTBP shows a similar ∆Tm to EcTBP, whose function as a TBP was proven (Figure 6D). Although to a lesser extent, the ∆Tm observed in OsTBP is also significant, showing increased protein stability upon thiamin addition. We conclude that thiamin binds tightly to EcTBP, FeTBP, and OsTBP.

## Discussion

Based on the report of the partial amino acid sequence of buckwheat TBP [28], we applied a bioinformatic approach to find plant genes encoding TBPs and identified the genes of putative binding proteins from *F. esculentum* (buckwheat, a dicot pseudocereal belonging to the family of *Polygonaceae*), *O. sativa* (rice, *Poaceae*), and *Z. mays* (maize, *Poaceae*). These proteins contained conserved structural motifs typical of seed storage proteins, such as the cupin conserved in the N- and C-terminal domains [29]. Notably, phylogenetic analysis showed a divergence among the putative TBPs, suggesting diverse evolutionary origins. This finding aligns with previous studies that highlight the heterogeneity of plant TBPs in terms of molecular mass, subunit structures, and biochemical properties [22].

*In silico* docking analysis supported the structural interaction of thiamin with the putative TBPs. *E. coli* TBP was used as a positive control given its proven function as a TBP. The residues surrounding thiamin predicted by the EcTBP docking model developed in this study are the same as the ones reported in the literature [37]. These correspond to Trp-193, Tyr-23, Trp-276, Ser-214, Tyr-211, Thr-158, Ser-157, Gly-56, and Asp-55 (Figure S2). This strengthens the prediction capabilities of our model. Based on this, and comparing the BEs of EcTBP with the ones of the putative plant TBPs, OsTBP and FeTBP have similar BEs and Ki values, while ZmTBP has a higher BE value. The objective of docking is to find the most stable receptor–ligand complex with optimized geometry and lower BE [36]. While all putative TBPs exhibited multiple binding pockets for thiamin, preferential binding was observed in a single pocket characterized by lower BEs and inhibitory constants. A lower BE indicates that thiamin has a stronger affinity for the TBP, as the interaction is energetically more favorable [36]. Similarly, a lower Ki reflects higher affinity, as Ki is inversely proportional to the binding affinity [36]. Furthermore, the conserved interaction between thiamin and a serine residue across all species underscores the significance of this residue in facilitating thiamin recognition and binding.

This *in silico* analysis confirmed the need to further investigate these putative TBPs, and therefore, we proceeded to *in vivo* expression studies in yeast, rice callus, and tobacco leaves to assess the functional relevance of the putative TBPs. In yeast, the overexpression of TBPs resulted in no significant change in thiamin concentration, likely due to a strict regulation on thiamin homeostasis. It is well documented that thiamin concentration in yeast is tightly regulated, wherein low thiamin levels activate transcription, while high levels exert repressive effects [41], a phenomenon known as the yeast *THI* regulatory system [42–44]. This regulation occurs predominantly at the transcriptional level [44] as part of yeast energy-saving strategy [45,46]. Additionally, in *Saccharomyces cerevisiae*, the activity of TBPs and thiamin transporters is repressed by excess thiamin [47]. Hence, the lack of impact from the expression of foreign TBPs on the thiamin concentration in yeast may be attributed to the tight feedback regulation of thiamin homeostasis, operating both at the transcriptional level and through the repression of TBPs and thiamin transporter activity under an excess of thiamin. This suggests that the yeast regulatory mechanisms prioritize maintaining optimal thiamin levels, preventing significant changes despite the overexpression of TBPs.

Notably, transient expression of TBPs in tobacco leaves and stable expression in rice callus resulted in a significant increase in thiamin concentration compared with the expression of thiamin biosynthetic genes alone (TTT), suggesting a potential role for TBPs in serving as a sink for thiamin *in planta*. However, the co-expression of TBPs with TTT did not increase the accumulation of thiamin, suggesting the existence of a currently unknown regulatory mechanism at the protein level, since we observed no correlation between gene expression and thiamin concentration in rice callus (Figure S3). While thiamin biosynthesis is known to be tightly regulated in plants, current understanding primarily revolves around end-product feedback regulation mediated via riboswitch control present in the 3′-UTR of *THIC* pre-mRNA [48–50]. In this regulatory mechanism, TDP binds to the riboswitch residues, forming an unstable splice variant. Additionally, plants can modulate thiamin biosynthesis by controlling other biosynthetic genes such as *THI1*, *TH1,* and *TPK,* although the specific regulatory mechanisms and roles in facilitating the accumulation of B1 vitamers and intermediates remain largely unexplored [48,51,52].

Drawing parallels with regulatory mechanisms governing other vitamin metabolism in plants, it is evident that the regulation of vitamin metabolism is complex, and transcriptional, translational, and post-translational events may occur, involving various components including protein–protein interactions and transporters [53]. For example, ζ-carotene isomerase activity is post-translationally regulated via redox changes causing conformational alterations [38], while pyridoxal phosphate synthase 1.2 (PDX1.2) forms a complex with its cognate pyridoxine biosynthesis enzyme pyridoxal phosphate synthase 1.3 (PDX1.3) to regulate vitamin B6 biosynthesis [39]. Other evidences also highlight the intricate regulatory networks governing vitamin metabolism. It is the case of mitochondrial accessory proteins, such as adrenodoxin and adrenodoxin reductase, that were found to interact with vitamin B7 synthase BIO2 *in vitro* [54]. This illustrates how little is still known about the regulatory mechanisms governing thiamin biosynthesis and metabolism in plants, especially in crops.

In both docking analysis and *in vivo* expression studies, rice and buckwheat TBPs were the most effective. For this reason, they were chosen to confirm their function through a thermal shift assay. The binding of thiamin to recombinant FeTBP and OsTBP was confirmed by the ligand-induced increase in protein stability, confirming the function of OsTBP and FeTBP as TBPs.

In this study, we identified and characterized TBPs previously unknown in plants. Future research could focus on elucidating the specific regulatory mechanisms governing thiamin biosynthesis and metabolism in plants, including the role of TBPs in thiamin storage and distribution within plant tissues. This study provides valuable insights into the potential use of TBPs as molecular targets for enhancing thiamin content in biofortified crops.

## Materials and methods

### Identification of plant TBPs and phylogenetic analysis

The partial amino acid sequence of the TBP from *F. esculentum* [28] served as the initial query in a BLASTp search against its complete proteome. This step resulted in the identification of FA02 protein as the putative *F. esculentum* protein. The FA02 protein sequence was then used as a query in a tBLASTn search against the *F. esculentum* genome. This enabled us to identify the genomic region containing the putative TBP coding sequence. With *F. esculentum* putative TBP sequence (*FeTBP*), we extended our analysis to other plant species and conducted BLASTn searches against the complete genomes of rice and maize. Alignments were made with MUSCLE [30] and visualized in Jalview [31]. The presence of conserved domains was evaluated in the putative ortholog proteins sequences using Pfam [55] and ScanProsite [56].

For the phylogenetic analysis, the full-length protein sequence from *F. esculentum* was used as a query to run Blastp to identify orthologs of FeTBP protein. The full-length amino acid sequences were aligned in MEGA X [57] using MUSCLE software [30]. The WAG model was selected, and the phylogenetic relationship was inferred using the maximum-likelihood method. The maximum-likelihood tree was evaluated with 1 000 bootstrap replicates. Phylogenetic trees were visualized using FigTree v1.4.2.

### Docking

For binding analysis, the structures of OsTBP (Uniprot code – Q6ESW6), FeTBP (Uniprot code – O23878), ZmTBP (Uniprot code – Q946V2), and *E. coli* TBP (EcTBP, Uniprot code – P31550) were downloaded from the SwissProt Protein Database [58]. The 2D structure of the ligand thiamin was geometrically optimized to a 3D model using ACD/ChemSketch software version 2020.1.1 (Advanced Chemistry Development, Inc., Toronto, ON, Canada). The charges of both the binding protein and ligand were adjusted by AVOGADRO 1.2.0 [59]. For the detection of thiamin-binding pockets and determination of the interaction energies associated with thiamin binding, a molecular docking analysis was performed by AutoDock version 4 (AutoDock suite) [60]. The whole proteins were covered by a grid (EcTBP: 31.989, 31.527, 0.375; FeTBP: −16.713, 60.458, −12.851; OsTBP: −12.966, 58.859, −12.193; ZmTBP: −12.807, 58.798, −12.286) with a space of 0.375 between grid points. The Lamarckian genetic algorithm was used as the docking search method, with the generation of 100 conformations, an initial population size of 300, a maximum number of evaluations of 25,000,000, and a maximum number of generations of 27,000. Ligand–protein docking was visualized in PyMOL (The PyMOL Molecular Graphics System, https://www.pymol.org/) [61].

### Gene cloning and vector construction

**Figure 7:**
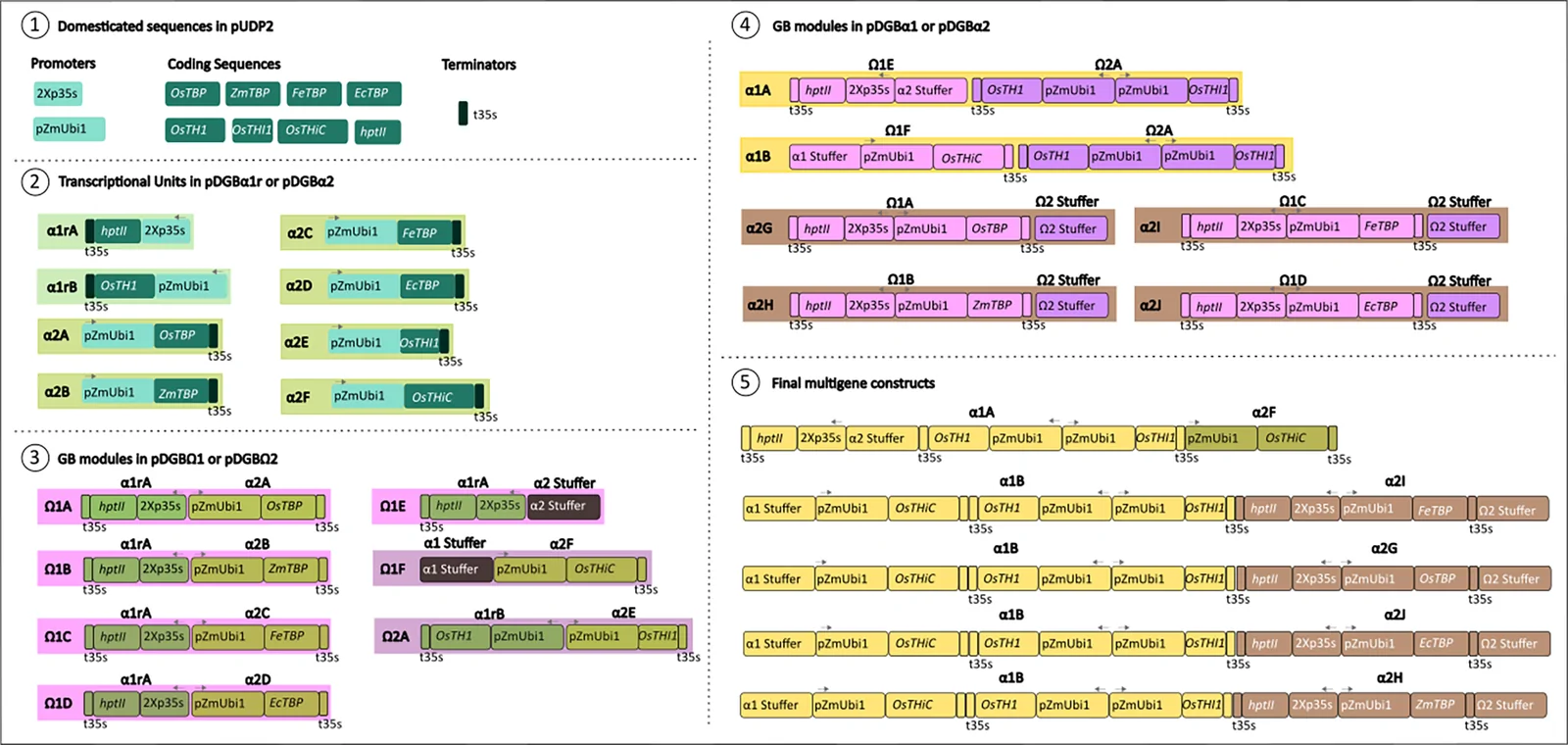
Schematic representation of GoldenBraid cloning path for constructs assembly. (**A**) Standard parts as promoters (2x35S and pZmUbi1), coding sequences (*OsTBP*, *ZmTBP*, *FeTBP*, *EcTBP*, *OsTH1*, *OsTHI1*, *OsTHIC,* and *htpii*), and terminators (t35S) were cloned into pUDP2 flanked by *Bsa*I cleavage sites. (**B**) Standard parts were assembled using level α plasmids (pDGB3α1r and pDGB3α2). Two transcriptional units were assembled in pDGBα1r: 2x35S-*hptII*-t35S (**α1rA**) and ZmUbi1-*OsTH1*-t35S (**α1rB**) Six transcriptional units were assembled in pDGBα2, with ZmUbi1 as promoter and t35S as terminator and *OsTBP* (**α2A**), *ZmTBP* (**α2B**), *FeTBP* (**α2C**), *EcTBP* (**α2D**), *OsTHI1* (**α2E**), or *OsTHIC* (**α2F**). **(C)**Two transcriptional units assembled in complementary α plasmids were used as entry vectors for a subsequent level Ω binary assembly. The *hptII* transcriptional unit (**α1rA**) was assembled with and *OsTBP* (**α2A**), *ZmTBP* (**α2B**), *FeTBP* (**α2C**), *EcTBP* (**α2D**), *OsTHIC* (**α2F**) transcriptional units in pDGBΩ1, creating **Ω1A**, **Ω1B**, **Ω1C**, **Ω1D**, **Ω1E,** and **Ω1F**, while *OsTH1* transcriptional unit (**α1rB**) was assembled with *OsTHI1* (**α2E**) in pDGBΩ2 (**Ω2A**). **(D & E)** Constructs assembled using complementary Ω plasmids were used as entry vectors for a subsequent level α binary assembly. Levels α and Ω were alternated until the final constructs were achieved. Whenever necessary, stuffers (sequences need for recombination between different plasmid type) were used. Flags represent the final constructs used in the study.

For overexpression in rice callus and *Nicotiana benthamiana* (tobacco) transient expression, the Golden Braid (GB) method was applied [62]. Domestication of the GB parts was done by removing the *Bsm*BI/*Esp*3I and *Bsa*I restriction sites and adding four nucleotide flanking overhangs to provide specificity to the part type, according to the GB protocol and website [62]. The predicted DNA sequences of *FeTBP*, *ZmTBP*, *OsTBP,* and *EcTBP* were retrieved from the National Center for Biotechnology Information [63]. *OsTBP, OsTH1*, *OsTHI1,* and *OsTHIC* were amplified from *O. sativa* japonica cv. Nipponbare cDNA with primers designed using the GB domesticator tool (https://gbcloning.upv.es/). *FeTBP*, *ZmTBP*, and *EcTBP* coding sequences were synthesized using IDT DNA eBlocks service (Table S2). The promoters, coding sequences, and terminators were inserted into pUDP2 vector using the standard GB reaction protocol [64]. The desired transcriptional units (TUs) were generated in pDGBα1r and pDGBα2 destination vectors, culminating in the generation of final multigene constructs through site-specific recombination [64]. The cloning steps are described in Figure 7.

For yeast overexpression assays, *OsTBP*, *FeTBP*, *ZmTBP,* and *EcTBP* were amplified from pUDP2, cloned into Gateway pDONR221 vector (Thermo Scientific, Waltham, Massachusetts, EUA) (primers depicted in Table S3) and recombined with pAG416 yeast expression vector (harboring GAL1 promoter and CYC1 terminator), using Gateway LR Clonase Enzyme Mix kit (Thermo Fisher Scientific), according to the manufacturer’s guidelines. For protein expression, pDONR221- *FeTBP*, pDONR221- *OsTBP,* and pDONR221- *EcTBP* were recombined with pDEST17 (containing a His Tag (6x)), via LR reaction.

Verification of the final constructs was performed by Sanger sequencing using a promoter forward primer, a terminator reverse primer, and an internal primer when needed for longer length parts.

### Strains and growth media

The *S. cerevisiae* wild-type strain BY4741 was used to perform TBP overexpression studies. Cells were grown in synthetic medium (20 g/L glucose, 6.7 g/L YNB, and 19 g/L agar) lacking amino acids (SD medium) with the exception of arginine 20 mg/L, isoleucine 30 mg/L, lysine 30 mg/L, methionine 20 mg/L, phenylalanine 50 mg/L, threonine 200 mg/L, tyrosine 200 mg/L, valine 150 mg/L, adenine 100 mg/L, and leucine 100 mg/L. BL21 (DE3) and DH5α *E. coli* strains were used for protein expression and plasmid purification, respectively, and grown in Luria-Bertani broth (LB) [65].

### Yeast overexpression assays

Lithium-Acetate method [66] was used to transform yeasts. The yeasts transformed with the pAG416 empty vector were used as negative control, while the ones transformed with *EcTBP* were used as positive control. Yeasts were grown over night, and the cultures were diluted with SD medium and OD_600_ = 0.2. Thiamin (100 mg/mL) was added to the medium, and cells were grown until OD_600_ = 1.2. Cultures were centrifuge and washed with water 3 times before HPLC-MS analysis.

### Plant material and transformation

Following established methods [67], the above-described binary vectors were introduced in *Agrobacterium tumefaciens* strain LBA4404. Scutellum-derived rice (*O. sativa* L. spp japonica cv. Nipponbare) calli were transformed by *Agrobacterium* as described by Upadhyaya et al. [68], and transformants were selected with 1 µl/mL hygromycin. To develop highly embryogenic tissues with a large number of somatic embryos, calluses were maintained in embryogenic induction medium [69] for one month. The presence of the hygromycin B resistance gene (*hpt*II) was verified using PCR amplification (Table S3). Three positive independent lines were stored at −80°C and assessed for each transgene.

### Transient expression in tobacco leaves

Transient expression in *N. benthamiana* was performed according to the method described by Sparkes et al. [70]. Leaves of 5-week-old tobacco grown under a 16-h light/8-h dark regime were transformed with Agrobacterium LBA4404. After 4 and 5 days, the infected areas of tobacco leaves were examined and collected for gene expression analysis. Based on the gene expression analysis, the samples collected at 5 Days after infection were analyzed for thiamin content.

### Thiamin extraction and quantification

The homogenized samples (100 mg each) were extracted with 1 mL of 50 mM phosphate buffer containing thiamin-d_3_ as an internal standard. The extract was vortexed for 10 s and stored for 2 h at 4°C. Then, it was purified with an Amicon 3 KDa centrifugal filter (15,900 rcf. at 4°C for 20 min) before loading to a liquid chromatography coupled with tandem mass spectrometry system (LC-MS). The LC-MS was a Waters ACQUITY UPLC and an Applied Biosystems API 4000 MS equipped with an electrospray ionization source. The extracted thiamin was separated on a Waters ACQUITY UPLC HSS T3 Column (2.1 mm X 150 mm, 100 Å, 1.8 µm) equipped with a Waters ACQUITY UPLC HSS T3 VanGuard Pre-column (2.1 mm X 5 mm, 100 Å, 1.8 µm) maintained at 45℃.

### RT-qPCR analysis

The mRNA of rice callus and tobacco leaves was extracted using Trizol reagent (Invitrogen), followed by DNAse treatment (ThermoFisher Scientific) and a cleanup step using GeneJET plant RNA purification kit (ThermoFisher Scientific) according to Strobbe et al. [16]. RNA (1 μg) was converted to cDNA using Bio-Rad iScript cDNA synthesis kit (containing oligo dT and random primers). qPCR was conducted on these cDNA samples using qPCR primers, as shown in Table S4. qPCRBIO SyGreen Mix with Fluorescein (Sopachem) was utilized in the reaction mix. A real-time quantitative PCR run comprised an initial denaturation at 95°C for 3 min and 40 cycles of denaturation at 95°C for 5 s, annealing for 20 s**,** and extension at 60°C for 6 s. Data analysis and normalization were performed using the qBASE software, based on the 2^–ΔΔCt^ method [71]. For rice callus gene expression analysis, ten candidate genes were selected for qPCR analysis (Table S4). The reference genes *Os25S* (LOC_Os09g01140) and *OseiE-4a* (LOC_Os02g05330) were selected for normalization based on geNorm software evaluation (Figure S4) [72]. Normalization was obtained by amplifying tobacco reference genes F-BOX (At5g15710) and L23 (At2g39460) for gene expression analysis in tobacco leaves.

### Protein overexpression and isolation

The plasmids pDEST17-*FeTBP*, pDEST17- *OsTBP,* and pDEST17- *EcTBP* and the empty vector were transformed into BL21 (DE_3_) *E. coli*. The cells were grown at 37°C in LB medium containing ampicillin (100 μg/mL) until reaching an OD_600nm_=0.6. Expression was induced by 0.1% L-Arabinose at 16°C for 16 h. Cells were harvested by centrifugation at 4000x***g***, 4°C for 15 min. Harvested cells were resuspended in 5 mL of lysis buffer (25 mM Tris, 300 mM NaCl, 5% glycerol, 1 mM PMSF (phenylmethylsulfonyl fluoride), 1 mg/mL lysozyme, DNAse (10 μg/mL) and 100 mM MgCl_2_), and incubated on ice for 30 min. Subsequently, the suspension was sonicated (60% amplitude; 10 cycles (30 s ON/30 s OFF), and the soluble fraction was obtained by centrifugation at 10,000x***g***, 4°C for 20 min. Proteins were purified through affinity chromatography using Ni-NTA columns. The lysate obtained was loaded onto the pre-equilibrated Ni-NTA column with base buffer (25 mM Tris, 300 mM NaCl, and 5% glycerol). The column was washed three times with 2 mL of washing buffer (25 mM Tris, 300 mM NaCl, 5% glycerol, 20 mM Imidazole, pH 8), and the protein was eluted with elution buffer (50 mM Tris, 300 mM NaCl, 2.5% glycerol, 500 mM imidazole, pH 8). Protein purity was assessed by sodium dodecyl sulfate-polyacrylamide gel electrophoresis analysis. Protein concentration was determined by the Bradford assay using bovine serum albumin as standard.

### Thermal shift assay

To monitor the thermal denaturation of the recombinant proteins upon thiamin binding, a thermal shift assay was performed using SPYRO Orange dye (Sigma S5692) in a LightCycler 480 (Roche) system. The reactions were conducted in a 96-well plate in a total volume of 10 μL. Each reaction was performed in 1 x PBS buffer. SPYRO Orange dye (5000x stock) was added to a final concentration of 5x as well as 500 μM of thiamin, and 5 mM EcTBP, 3 mM FeTBP, 3 mM OsTBP, or buffer. The assay plate was kept at 25°C for 2 min, followed by ramping up in increments of 0.05°C/s to a final temperature of 95°C. The protein’s melting temperature (Tm) was determined by performing nonlinear fitting of the dataset to a Boltzmann sigmoidal curve using GraphPad software.

### Statistical analysis

Differences in means are indicated for datasets in which *P*-values below 0.05 are considered significant, and *P*-values below 0.01 are considered very significant. The statistical analysis was performed using GraphPad8. For thiamin concentration in yeast, rice callus, and tobacco leaves, three independent samples were assessed. Data were compared with positive control values (EcTBP and TTT) by one-way ANOVA followed by Tukey’s post hoc test. For melting temperature analysis, the average melting temperature difference from four independent experiments was calculated.

## Supplementary material

online supplementary material 1.

## Data Availability

All data from this study are included in the article.

## Abbreviations

BE: Binding energy
GB: Golden braid
LB: Luria-Bertani broth
TBP: thiamin-binding protein
TDP: thiamin diphosphate
TMP: thiamin monophosphate
