# Supplementary material for: In silico, in vitro, and in vivo characterization of thiamin-binding proteins from plant seeds
Source: Biochem J. 2025 Feb 5;482(4):BCJ20240429. doi: 10.1042/BCJ20240429 (PMC12400320; doi:10.1042/BCJ20240429)
Supplement: online supplementary material 1. [file bcj-482-4-BCJ20240429-s001.docx]

**Supplementary Data**

***In silico*, *in vitro* and *in vivo* studies reveal thiamin binding proteins from plant seeds** *(max 20 words)*

Maria Faustino^1,2,a^, Simon Strobbe^1,a^, Raul Sanchez-Muñoz^1^, Da Cao^1^, Ratnesh C. Mishra^1^, Tiago Lourenço^2^, M. Margarida Oliveira^2*^, Dominique Van Der Straeten^1*^.

^1^ Laboratory of Functional Plant Biology, Department of Biology, Ghent University, K. L. Ledeganckstraat 35, B-9000 Gent, Belgium.

^2^ Laboratory of Plant Functional Genomics, Instituto de Tecnologia Química e Biológica António Xavier, Universidade Nova de Lisboa, 2780-157, Oeiras, Portugal.

^a^Present address: University of Geneva, Quai E. Ansermet 30, 1211 Geneva, Switzerland.

^*^Correspondence:

Dominique Van Der Straeten: [dominique.vanderstraeten@ugent.be](mailto:dominique.vanderstraeten@ugent.be); M. Margarida Oliveira: mmolive@itqb.unl.pt

**Supplementary** **Figures**


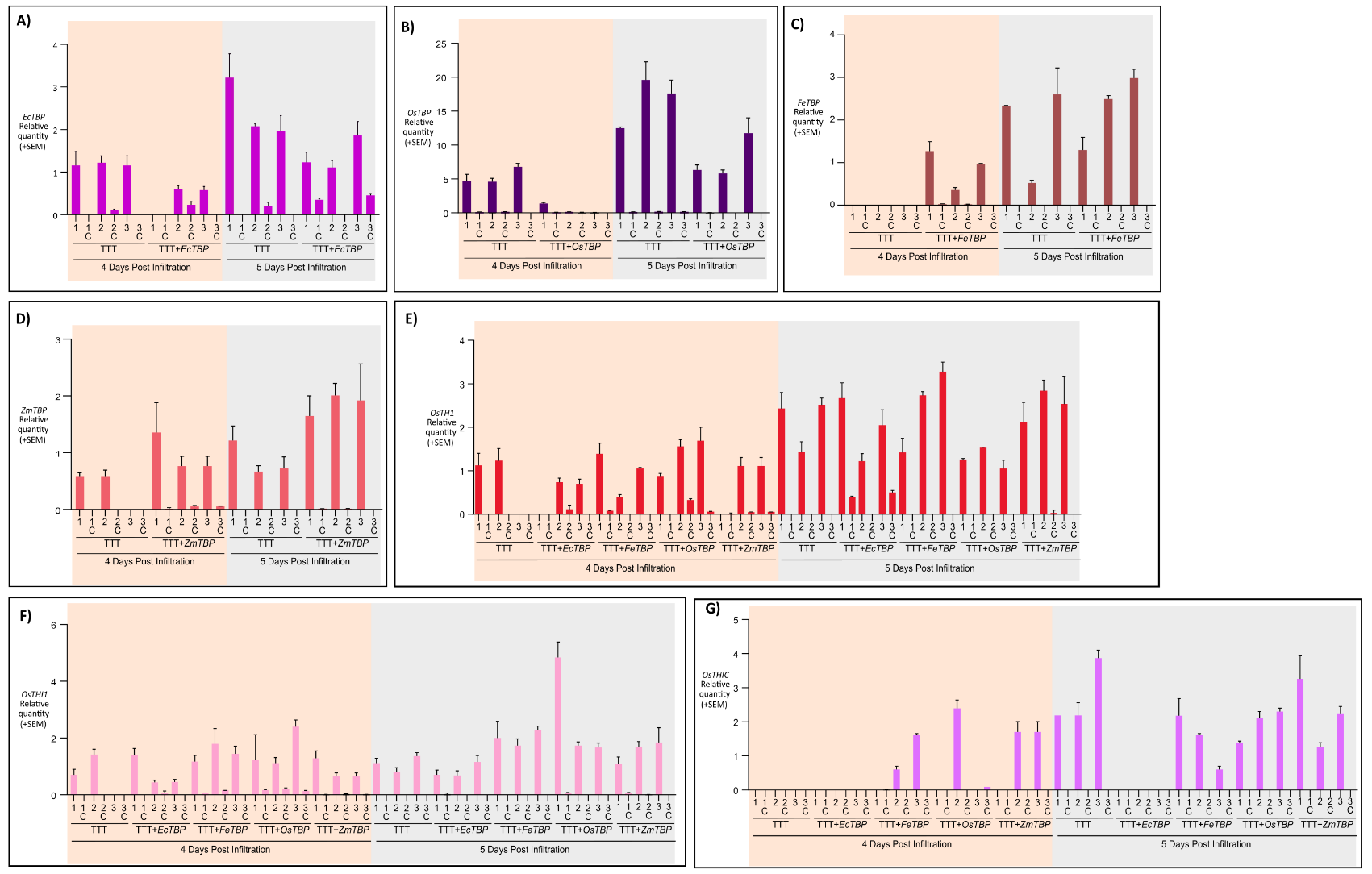


**Figure S1.** Transient gene expression in tobacco of *OsTH1*, *OsTHI1*, *OsTHIC, EcTBP*, *FeTBP*, *OsTBP,* and *ZmTBP* relative to *F-BOX* (At5g15710) and *L23* (At2g39460) housekeeping genes in tobacco leaves 4 and 5 days afterAgrobacterium infiltration. Relative gene expression of **(A)** *EcTBP*, **(B)** *OsTBP*, **(C)** *FeTBP*, **(D)** *ZmTBP*, **(E)** *OsTH1*, **(F)** *OsTHI1,* and **(G)** *OsTHIC*.


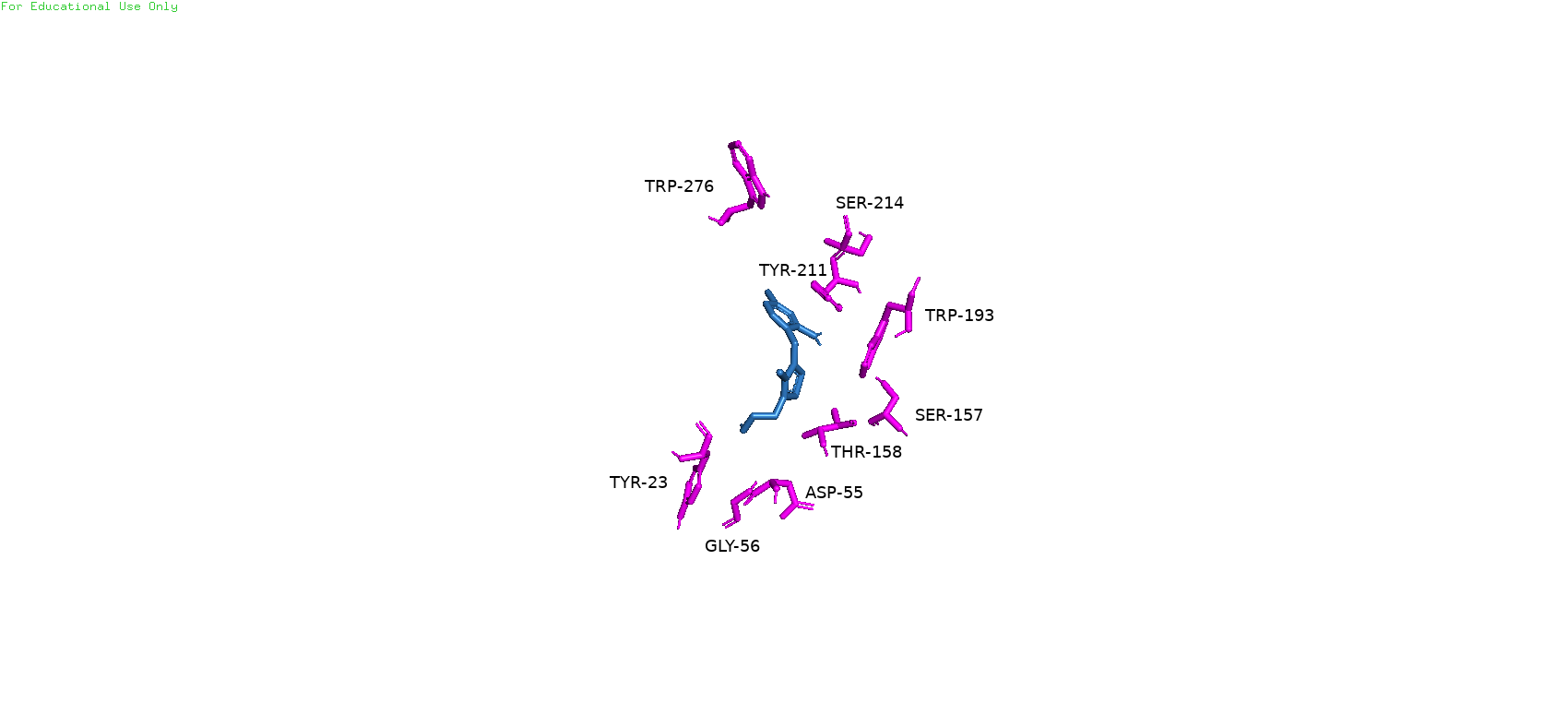


**Figure S4.** Amino acid residues surrounding thiamin generated by AutoDockTools to the EcTBP structure with thiamin bound.

**
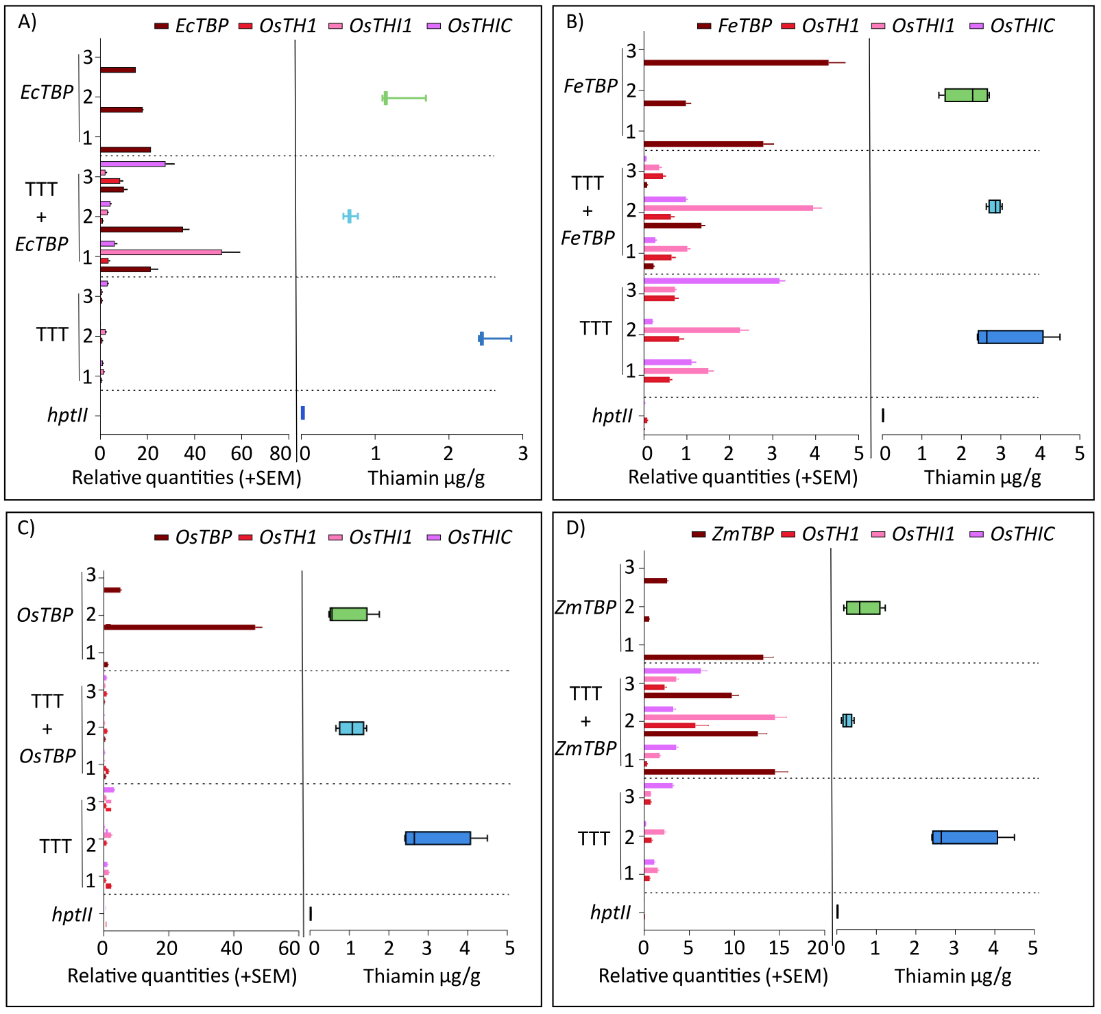
**

**Figure S3. Gene expression in rice callus relative to *Os25S* and *OseiE-4a* housekeeping genes and thiamin concentration. (A)** Thiamin concentration and relative gene expression of *OsTH1*, *OsTHI1*, *OsTHIC,* and *EcTBP* in three independent rice callus lines expressing *EcTBP* and coexpressing *TTT* and *EcTBP*. **(B)** Thiamin concentration and relative gene expression of *OsTH1*, *OsTHI1, OsTHIC,* and *FeTBP* in three independent rice callus lines expressing *FeTBP* and coexpressing *TTT* and *FeTBP*. **(C)** Thiamin concentration and relative gene expression of *OsTH1*, *OsTHI1*, *OsTHIC,* and *OsTBP* in three independent rice callus lines expressing *OsTBP* and coexpressing *TTT* and *OsTBP* and thiamin concentration. **(D)** Thiamin concentration and relative gene expression of *OsTH1*, *OsTHI1*, *OsTHIC,* and *ZmTBP* in three independent rice callus lines expressing *ZmTBP* and coexpressing *TTT* and *ZmTBP*.


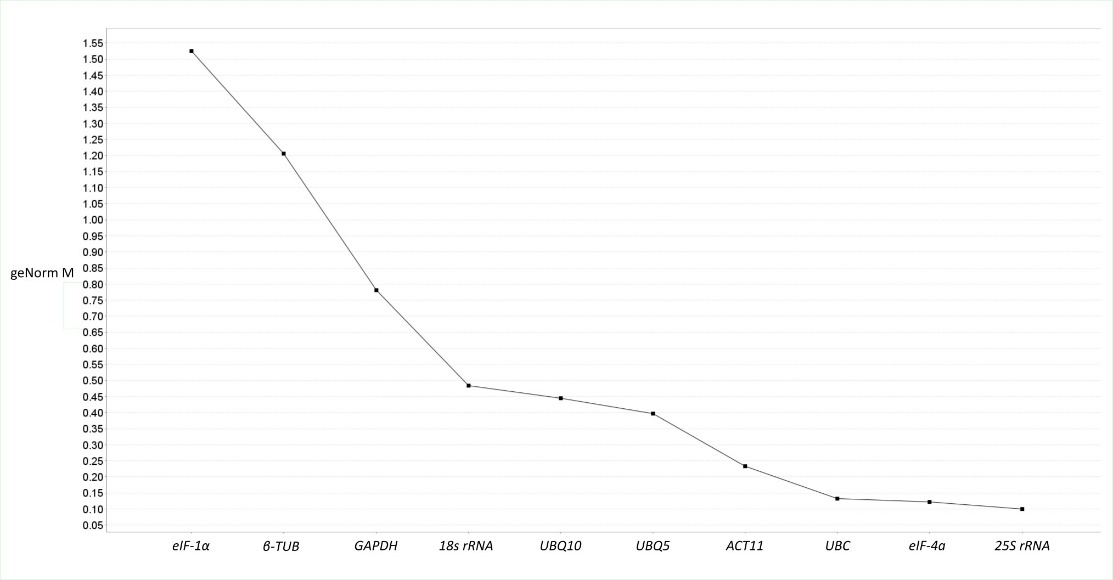


**Figure S4**. Measurement (M) values of ten reference genes were calculated by geNorm and ranked across all the samples. A higher M value means lower stability, while a lower M value implies more stable gene expression. eIF-1α (LOC_Os03g08020), eukaryotic elongation factor 1-alpha; β-TUB (LOC_Os01g59150), beta-tubulin; GAPDH (LOC_Os04g40950), glyceraldehyde-3-phosphate dehydrogenase; 18s rRNA (LOC_Os09g00999), 18S ribosomal RNA; UBQ10 (LOC_Os02g06640), ubiquitin 10; UBQ5 (LOC_Os01g01010), ubiquitin 5, *ACT11* (LOC_Os03g50885), actin 11; UBC (LOC_Os02g42314), ubiquitin-conjugating enzyme E2; eIF-4a (LOC_Os02g05330), eukaryotic initiation factor 4a; 25S rRNA (LOC_Os09g01140), 25S ribosomal RNA

**Supplementary Tables**

**Table S1.** Sequences used in phylogenetic studies of putative (Arabidopsis thaliana, *Asparagus officinalis*, *Brassica rapa*, *Fagopyrum esculentum*, *Oryza sativa*, *Sorghum bicolor*, *Triticum aestivum,* and *Zea mays)* and known (*Escherichia coli* and *Sesamum indicum*) thiamin binding proteins.

| > Arabidopsis thaliana, AT1G03890, RmlC-like cupins superfamily protein  MHKLLFSLLSVVSLSFLLFFHGAEARQREAPFPNACHFSQINSLAPAQATKFEAGQMEVWDHMSPELRCAGVTVARITLQPNSIFLPAFFSPPALAYVVQGEGVMGTIASGCPETFAEVEGSSGRGGGGDPGRRFEDMHQKLENFRRGDVFASLAGVSQWWYNRGDSDAVIVIVLDVTNRENQLDQVPRMFQLAGSRTQEEEQPLTWPSGNNAFSGFDPNIIAEAFKINIETAKQLQNQKDNRGNIIRANGPLHFVIPPPREWQQDGIANGIEETYCTAKIHENIDDPERSDHFSTRAGRISTLNSLNLPVLRLVRLNALRGYLYSGGMVLPQWTANAHTVLYVTGGQAKIQVVDDNGQSVFNEQVGQGQIIVIPQGFAVSKTAGETGFEWISFKTNDNAYINTLSGQTSYLRAVPVDVIKASYGVNEEEAKRIKFSQQETMLSMTPSSSS |
| --- |
| >*Asparagus officinalis*, LOC109836330, 11S globulin seed storage protein 2-like  MAEAIGILVDLMRKIQRKEERGVLVNVEEEMRTIVPDEEQEGRDVRVNKEMGNNGNETNGEFCSIRLLQAIDRIGDADVFSKKAGWLNKITEDKLPLLRLVDLSAEKGSLQPNAFLVPHWSVNAHSIIYVTQGEGHLQVVDNQGRGVFNVNIKQGQLTVVPQYYACMIRAGSSGINWVTFMTSSQPMRTPIVGRLSTFTALPLQPMANSYRIPITQALELKFNRDHDEMLFPPTTTSYQTTE |
| >*Brassica rapa*, A09p082400, 12S seed storage protein  MSTKLILSFSLCLMVLSCSAQLLPWRKGQRSRPHRGHQQFHHQCDVQRLTASEPSRRVRSEAGVTEIWDNDTPEFRCAGFVAVRVVIQPGGLLLPSYSNAPYITFVEQGRGVQGVVVPGCPETFQSESEFEYPQSQRDQRSRQSESEESSRGDQRTRQSESEEFSRGDQRTRQSESEEFSRGDQRTRQSESEEFSRGDQRTRQSESEEFSRGDQHQKIFRIRDGDVIPSPAGVVQWTHNDGDNDLISITLYDANSFQNQLDGNVRNFFLAGQSKQSREDRRSQRQTREEGSDRQSRESDDDEALLEANILTGFQDEILQEIFRNVDQETISKLRGDNDQRGFIVQARDLKLRVPEEYEEELQRERGDRKRGGSGRSNGLEQAFCNLKFKQNVNRPSRADVFNPRAGRINTVNSNNLPILEFIQLSAQHVVLYKNAILGPRWNLNAHSALYVTRGEGRVQVVGDEGRSVFDDNVQRGQILVVPQGFAVVLKAGREGLEWVELKNDDNAITSPIAGKTSVLRAIPVEVLANSYDISTKEAFRLKNGRQEVEVFLPFQSRDEKERERF |
| > *Escherichia coli*, thiB, thiamin ABC transporter periplasmic binding protein  MLKKCLPLLLLCTAPVFAKPVLTVYTYDSFAADWGPGPVVKKAFEADCNCELKLVALEDGVSLLNRLRMEGKNSKADVVLGLDNNLLDAASKTGLFAKSGVAADAVNVPGGWNNDTFVPFDYGYFAFVYDKNKLKNPPQSLKELVESDQNWRVIYQDPRTSTPGLGLLLWMQKVYGDDAPQAWQKLAKKTVTVTKGWSEAYGLFLKGESDLVLSYTTSPAYHILEEKKDNYAAANFSEGHYLQVEVAARTAASKQPELAQKFLQFMVSPAFQNAIPTGNWMYPVANVTLPAGFEKLTKPATTLEFTPAEVAAQRQAWISEWQRAVS |
| > Fagopyrum esculentum, FA02, 13S globulin seed storage protein 1  MSTKLILSFSLCLMVLSCSAQLLPWRKGQRSRPHRGHQQFHHQCDVQRLTASEPSRRVRSEAGVTEIWDNDTPEFRCAGFVAVRVVIQPGGLLLPSYSNAPYITFVEQGRGVQGVVVPGCPETFQSESEFEYPQSQRDQRSRQSESEESSRGDQRTRQSESEEFSRGDQRTRQSESEEFSRGDQRTRQSESEEFSRGDQRTRQSESEEFSRGDQHQKIFRIRDGDVIPSPAGVVQWTHNDGDNDLISITLYDANSFQNQLDGNVRNFFLAGQSKQSREDRRSQRQTREEGSDRQSRESDDDEALLEANILTGFQDEILQEIFRNVDQETISKLRGDNDQRGFIVQARDLKLRVPEEYEEELQRERGDRKRGGSGRSNGLEQAFCNLKFKQNVNRPSRADVFNPRAGRINTVNSNNLPILEFIQLSAQHVVLYKNAILGPRWNLNAHSALYVTRGEGRVQVVGDEGRSVFDDNVQRGQILVVPQGFAVVLKAGREGLEWVELKNDDNAITSPIAGKTSVLRAIPVEVLANSYDISTKEAFRLKNGRQEVEVFLPFQSRDEKERERF |
| >*Oryza sativa*, LOC_Os02g14600, Glutelin type-B 5  MATIAFSRFSICFCVLLLCHGSMAQIFSLGINPWQNPRQGGSRECRFDRLQAFEPLRKVRHEAGVTEYFDEKNEQFQCTGTLVIRRIIEPQGLLLPRYSNTPGLVYIIQGTGVLGLTFPGCPATYQKQFRHFGLEGGSQRQGKKLRDENQKIHQFRQGDVVALPSGIPHWFYNEGDTPVVALFVFDVNNNANQLEPRQKEFLLAGNNIEQQVSNPSINKHSGQNIFNGFNTKLLSEALGVNIEVTRRLQSQNDRRGDIIRVKNGLRLIKPTITQQQEQTQDQYQQIQYHREQRSTSKYNGLDENFCAIRARLNIENPNHADTYNPRAGRITNLNSQKFSILNLVQMSATRVNLYQNAILSPFWNINAHSLVYTIQGRARVQVVSNHGKAVFNGVLRPGQLLIIPQNYVVMKKAELEGFQFIAFKTNPNAMVNHIAGKNSVLRAMPVDVIANAYRISRQEARSLKNNRGEEIGAFTPRYQQQKIHQEYSNPNESETQEVI |
| > *Sesamum indicum*, LOC105178074, 2S albumin  MARFSACMVLIALIIVLLAAATTAEIVNPGRESQRCRQQIERQRLSSCREYLIDSSRPVMMEGGNQGRSWREEFPRCCEELERIDEQCRCQAVQQVVQQERQGGELQGRELQEMLQTAQSLPSLCRISPRYCQNMGEVAGPMF |
| > *Sorghum bicolor,* LOC8068650*,* glutelin type-B 5  MAAAASLSGKLLFPSSLCLCLLLLCCSGAGGAAASSSWGASRGGAARECGFDGKLEALEPRHKAQSEAGSVEYFSRFTEADRELTCAGLFAVRVVVDALGLVLPRYSNLHSLVYIAQGRGIIGFSFPGCQEETHHQQQYGYGYGYEHHHQRPDEHHKIHRFQQGDVVAMPAGAQHWLYNDGDTPLVAIYVFDTNNNINQLEPSMRKFLLAGGFSRGQPHFAENIFKGIDARFLSEALGVSMQVAEKLQSRREQRGEIVRVELEHGLHLLNPPPPSFPSLQDQYQHHQTCQRDNSRNICTMEVRHSVERLDQADVYSPGAGRITRLTSHKFPILNLIQMSAVRVDLYQDAILSPFWNFNAHSAMYTIRGCARVQVASDNGTTVFDGVLRAGQLLIIPQGYLVATKAQGEGFQYISFETNHNSMVSHIAGKNSLLSDLPVGVIASSYGVSMEEAAELKNSRKHELAVFTTPPGGSYDQGHVGSAQQ |
| > *Triticum aestivum*, TraesCS1D02G067100, cupin type-1 domain-containing protein  MAATSFASLSFYFCILLLCHSSMAQLFGMSFNPWQSSRQGGFRECTFNRLQASTPLRQVRSQAGLTEYFDEENEQFRCTGVFAIRRVIEPRGYLLPRYHNTHGLVYIIQGSGFAGLSFPGCPETFQKQFQKYGQSQSVQGQSQSQKFKDEHQKVHRFRQGDVIALPAGIVHWFYNDGDAPIVAIYVFDVNNYANQLEPRHKEFLFAGNYRSSQLHSSQNIFSGFDVRLLAEALGTSGKIAQRLQSQNDDIIHVNHTLKFLKPVFTQQREPESYPHTQYEEGQSQAKHTQGEQPQMGWSQAGPYPGCQPHAGQSHASQSTYGGWNGLEENFCDHKLSVNIDDPSRADIYNPRAGTITRLNSQTFPILNIVQMSATRVHLYQNAIISPLWNINAHSVMYMIQGHIWVQVVNDHGRNVFNDLLSPGQLLIIPQNYVVLKKAQRDGSKYIEFKTNANSMVSHIAGKNSILGALPVDVIANAYGISRTEARSLKFSREEELGVFAPKFSQSIFRSFPNGEEESS |
| > *Zea mays*, GRMZM2G174883, legumin 1  MAAAIVLSGQVRPLPSSLPLSLLLLLLLCCSGTSWGWSTSRGGAARECGFDGKLEALEPRHKVQSEAGSVQYFSRFNEADRELTCAGIFAVRVVVDAMGLLLPRYSNVHSLVYIVQGRGIIGFSFPGCQEETQQQQYGYGYGYGHHHHQHDHHKIHRFEQGDVVAMPAGAQHWLYNDGDAPLVAVYVFDENNNINQLEPSMRKFLLAGGFSKGQPHFAENIFKGIDARFLSEALGVSMHVAEKLQSRRDQRGEIVRVEPEHGFHQLNPSPSSSSFSFPSSQVQYQTCQRDVDRHNVCAMEVRHSVERLDQADVYSPGAGRITRLTSHKFPVLNLVQMSAVRVDLYQDAIMSPFWNFNAHSAMYGIRGSARVQVASDNGTTVFDDVLRAGQLLIVPQGYLVATKAQGEGFQYIAFETNPDTMVSHVAGKNSVLSDLPAAVIASSYAISMEEAAELKNGRKHELAVLTPAGSGSYQQGQAGSAQQ |

**Table S2.** Sequence of the GoldenBraid parts synthesized using IDT DNA eBlocks^TM^ service.

| >*O. sativa* codon optimized *E. coli TBP* (IDT DNA)_Patch 1  AAA GCGC CGT CTC G CTCG A ATG TCC GCG CCA GCC GTT GCA GTC ACT GCG CCG GTG TTT GCA AAG CCC GTG CTC ACA GTC TAC ACT TAT GAC TCG TTC GCT GCG GAT TGG GGA CCC GGC CCC GTG GTG AAG AAG GCT TTC GAA GCG GAT TGT AAC TGT GAA TTG AAG CTC GTG GCG TTG GAG GAC GGA GTG TCC CTT CTT AAC CGG CTT AGG ATG GAG GGC AAA AAT TCC AAA GCC GAC GTT GTT CTT GGG CTG GAT AAC AAT TTG TTG GAT GCG GCG AGT AAA ACG GGG TTG TTC GCT AAA TCC GGC GTT GCG GCA GAT GCG GTG AAC GTG CCA GGC GGT TGG AAC AAT GAC ACT TTC GTC CCC TTC GAT TA C GAGACG GCGC AAA |
| --- |
| >*O. sativa* codon optimized *E. coli TBP* (IDT DNA)_Patch 2  AAA GCGC CGT CTC G AT TAT GGT TAC TTT GCT TTC GTC TAC GAC AAA AAT AAA TTG AAA AAT CCT CCG CAA TCA CTT AAA GAG CTG GTG GAG TCC GAT CAA AAC TGG CGC GTT ATA TAC CAA GAT CCA AGG ACT AGT ACT CCC GGT CTT GGG TTG CTT TTG TGG ATG CAG AAA GTC TAT GGT GAC GAT GCC CCT CAG GCC TGG CAG AAG CTG GCT AAG AAG ACT GTC ACC GTT ACT AAA GGT TGG TCT GAA GCA TAC GGA CTC TTC CTT AAA GGG GAA AGT GAT CTT GTG CTC TCG TAC ACG ACA TCG CCA GCC TAT C C GAGACG GCGC AAA |
| >*O. sativa* codon optimized *E. coli TBP* (IDT DNA)_Patch 3  AAA GCGC CGT CTC G TAT CAT ATC TTG GAG GAG AAG AAA GAT AAT TAT GCC GCT GCC AAC TTT TCC GAA GGG CAC TAT TTG CAG GTT GAG GTC GCA GCA AGG ACC GCG GCC AGT AAA CAG CCG GAG CTG GCG CAA AAA TTT TTG CAA TTT ATG GTG TCC CCA GCG TTT CAG AAC GCC ATC CCC ACG GGC AAC TGG ATG TAC CCA GTC GCT AAT GTG ACT CTC CCA GCG GGA TTT GAA AAA CTG ACC AAG CCG GCA ACC ACT CTT GAA TTT ACT CCC GCA GAA GTG GCT GCG CAA AGA CAA GCG TGG ATT TCT GAG TGG CAG AGA GCC GTT AGT AGA TAAGCTTTGAG C GAGACG GCGC AAA |
| >*O. sativa* codon optimized *F. esculentum TBP* (IDT DNA)_Patch 1  AAA GCGC CGT CTC G CTCG A ATG TCA ACA AAA CTG ATC CTG TCC TTT TCA CTC TGC TTG ATG GTC TTG TCA TGT TCC GCG CAA CTT CTC CCT TGG CGG AAA GGC CAG CGC AGC CGC CCT CAC CGG GGG CAT CAG CAG TTT CAC CAT CAG TGT GAC GTT CAA AGA CTG ACT GCT AGT GAA CCT TCT AGA CGG GTT CGG TCA GAG GCC GGA GTT ACA GAA ATA TGG GAC AAC GAT ACC CCA GAG TTC CGC TGT GCG GGG TTC GTT GCA GTT CGG GTC GTC ATT CAA CCT GGA GGA CTC TTG TTG CCG TCC TAT TCT AAC GCC CCG TAT ATT ACC TTT GTC GAA CAG GGG CGC GGG GTG CAG GGC GTT GTT GTG CCC GAGACG GCGC AAA |
| >*O. sativa* codon optimized *F. esculentum TBP* (IDT DNA)_Patch 2  AAA GCGC CGT CTC G TG CCG GGC TGC CCT GAG ACC TTC CAG AGT GAG TCT GAG TTC GAG TAC CCG CAG TCT CAA CGC GAT CAA CGC AGT CGC CAG TCA GAG TCT GAA GAA AGC TCT AGA GGT GAC CAG CGG ACT AGA CAG TCG GAA TCC GAG GAA TTC AGT CGC GGC GAC CAA CGG ACG CGG CAG TCC GAG AGC GAA GAA TTC AGT AGG GGA GAC CAG AGG ACG CGC CAA TCT GAG TCC GAG GAG TTT TCC AGA GGG GAT CAA AGG ACA AGG CAA TCT GAA AGC GAG GAG TTC TCT CGC GGG GAC CAA CAT CAG AAA ATA TTT AGG ATC CGC GAT GGG GAT GTC ATT CCC TCC CCA GC C GAGACG GCGC AAA |
| >*O. sativa* codon optimized *F. esculentum TBP* (IDT DNA)_Patch 3  AAA GCGC CGT CTC G CA GCA GGT GTT GTT CAA TGG ACT CAT AAC GAC GGC GAT AAT GAC CTG ATA AGT ATC ACT CTT TAT GAT GCC AAC AGC TTC CAA AAT CAA CTG GAT GGA AAT GTC CGG AAC TTC TTC CTC GCA GGA CAG TCT AAA CAG TCC AGG GAA GAT AGA CGG AGC CAA AGG CAG ACC CGG GAA GAA GGG AGC GAC AGA CAG AGC CGG GAG AGC GAC GAC GAC GAA GCC CTC CTC GAA GCG AAC ATC CTC ACT GGT TTT CAG GAT GAA ATA CTG CAG GAA ATT TTT AGA AAT GTC GAC CAG GAG ACA ATT AGT AAG CTG CGG GGG GAC AAC GAT CAG AGG GGA TTC AT C GAGACG GCGC AAA |
| >*O. sativa* codon optimized *F. esculentum TBP* (IDT DNA)_Patch 4  AAA GCGC CGT CTC G TC ATT GTC CAG GCG AGG GAT CTG AAA TTG CGG GTT CCC GAA GAA TAT GAG GAG GAA TTG CAA AGA GAA CGC GGA GAT CGC AAA CGG GGG GGT AGC GGT AGA TCC AAC GGG CTG GAG CAA GCC TTC TGT AAT CTG AAG TTC AAA CAG AAC GTG AAC AGG CCT TCG AGG GCA GAC GTT TTT AAC CCG CGG GCG GGC AGG ATC AAC ACG GTC AAT TCG AAC AAT TTG CCC ATT CTC GAA TTC ATC CAG CTC TCT GCG CAA CAT GTG GTG TTG TAC AAA AAT GCC ATA TTG GGG CCG AGG TGG AAC CTC AAC GCA CAT AGC GCA CT C GAGACG GCGC AAA |
| >*O. sativa* codon optimized *F. esculentum TBP* (IDT DNA)_Patch 5  AAA GCGC CGT CTC G CA CTG TAT GTT ACC CGG GGA GAG GGA AGA GTG CAG GTG GTC GGA GAT GAG GGT CGC TCA GTG TTC GAC GAC AAT GTG CAA AGA GGG CAA ATC CTG GTT GTG CCT CAG GGA TTC GCA GTT GTC CTG AAA GCA GGT AGA GAG GGT CTG GAG TGG GTC GAA TTG AAG AAT GAC GAT AAC GCA ATA ACG AGT CCT ATA GCT GGG AAG ACT AGC GTG CTT AGA GCG ATC CCC GTC GAA GTG CTT GCC AAT TCA TAC GAT ATT TCG ACT AAA GAA GCG TTC CGC TTG AAA AAT GGG AGA CAA GAG GTC GAA GTG TTC CTC CCT TTT CAG TCG CGC GAC GAA AAG GAG AGA GAG AGA TTT TAA GCTTTGAG C GAGACG GCGC AAA |
| >*O. sativa* codon optimized *Z. mays TBP* (IDT DNA)_Patch 1  AAA GCGC CGT CTC G CTCG A ATG GCC GCA GCT ATA GTC CTT AGT GGG CAA GTT AGG CCT CTT CCT TCT AGT CTG CCC CTC TCA CTG TTG TTG CTC TTG TTG CTT TGC TGT AGT GGA ACC TCC TGG GGG TGG TCG ACT TCA AGA GGT GGG GCT GCT CGC GAA TGC GGA TTC GAT GGC AAG CTG GAG GCG CTG GAA CCT AGA CAT AAA GTC CAA TCC GAG GCG GGG TCT GTG CAG TAC TTC AGC AGG TTT AAT GAG GCC GAC AGG GAG CTC ACC TGT GCA GGG ATA TTT GCA GTG AGA GTG GTG GTG GAT GCG ATG GGA CTT CTT CTC CCC AGG TAT TCA AAT GTG CAT TCA CT C GAGACG GCGC AAA |
| >*O. sativa* codon optimized *Z. mays TBP* (IDT DNA)_Patch 2  AAA GCGC CGT CTC G CA CTG GTG TAT ATT GTC CAG GGT CGG GGT ATC ATA GGA TTC TCA TTC CCT GGC TGC CAG GAG GAG ACA CAA CAA CAG CAG TAT GGA TAC GGG TAC GGG TAT GGG CAC CAT CAC CAC CAA CAC GAC CAT CAT AAG ATA CAC AGA TTC GAG CAG GGG GAC GTT GTC GCG ATG CCT GCC GGC GCG CAA CAC TGG CTT TAC AAC GAT GGT GAC GCG CCA CTT GTT GCC GTG TAT GTC TTC GAT GAG AAC AAC AAC ATA AAT CAA CTG GAG CCG TCT ATG CGC AAG TTC CTT TTG GCG GGG GGG TTC AGC AAA GGC CAA CCC CAT TTT GCA GAG AAT AT C GAGACG GCGC AAA |
| >*O. sativa* codon optimized *Z. mays TBP* (IDT DNA)_Patch 3  AAA GCGC CGT CTC G AT ATA TTC AAG GGT ATC GAC GCA AGG TTT CTC TCC GAG GCA TTG GGC GTG TCT ATG CAT GTC GCC GAG AAA CTT CAG TCT AGG AGG GAT CAG AGG GGA GAA ATC GTC AGA GTT GAG CCG GAG CAT GGT TTT CAC CAG CTT AAT CCG AGT CCC TCT AGT AGC AGT TTT TCG TTC CCT TCG TCA CAA GTC CAA TAC CAA ACG TGT CAA CGC GAC GTG GAC AGG CAC AAT GTC TGC GCA ATG GAG GTC CGC CAC TCC GTT GAG CGC CTC GAT CAA GCG GAC GTC TAC TCA CCA GGG GCG GGC CGG ATC ACA AGA TTG ACG AGC CAC AAG TTC CCG GT C GAGACG GCGC AAA |
| >*O. sativa* codon optimized *Z. mays TBP* (IDT DNA)_Patch 4  AAA GCGC CGT CTC G CG GTC CTT AAC CTG GTG CAA ATG TCT GCT GTC AGG GTC GAT CTG TAT CAA GAC GCC ATT ATG TCC CCC TTT TGG AAC TTC AAC GCT CAT TCT GCA ATG TAT GGG ATT AGA GGC TCT GCG AGA GTG CAA GTT GCG TCC GAT AAC GGA ACC ACG GTT TTC GAT GAC GTG CTT AGA GCC GGC CAG CTG CTG ATA GTG CCC CAG GGA TAC TTG GTT GCT ACC AAA GCT CAG GGG GAG GGG TTT CAG TAT ATT GCT TTC GAA ACA AAT CCT GAC ACT ATG GTG AGC CAC GTC GCA GGC AAA AAT AGT GTG TTG AGT GAC TTG CCC GCT GCA GTG ATA GCG TCT AGC TAT GCG ATA TCG ATG GAA GAG GCT GCA GAG TTG AAA AAT GGA CGG AAG CAC GAG CTG GCT GTG CTC ACG CCT GCG GGG TCA GGA TCG TAT CAA CAA GGA CAA GCT GGA TCG GCC CAG CAG TAG GCTTTGAG C GAGACG GCGC AAA |

**Table S3.** Primers used in the study.

| Primer pair | Sequence 5’ 🡪 3’ | Use |
| --- | --- | --- |
| 1 | GCGCCGTCTCGCTCGAATGGCAACTATTGCATTCTCTC | *OsTBP* amplification from cDNA |
|  | GCGCCGTCTCGCTCAAAGCTTAAATCACCTCTTGAGTCTCAC |  |
| 2 | GGGGACAAGTTTGTACAAAAAAGCAGGCTCCATGTCAACAAAACTGATCCT | *FeTBP* amplification from pUDP2 for GT cloning |
|  | GGGGACCACTTTGTACAAGAAAGCTGGGTATCCTCTCTCTCTCTAAAATT |  |
| 3 | GGGGACAAGTTTGTACAAAAAAGCAGGCTCCATGTCCGCGCCAGCCGTT | *EcTBP* amplification from pUDP2 for GT cloning |
|  | GGGGACCACTTTGTACAAGAAAGCTGGGTATTATCTACTAACGGCTCTCTGCC |  |
| 4 | GGGGACAAGTTTGTACAAAAAAGCAGGCTCCATGGCCGCAGCTATAGTCCT | *ZmTBP* amplification from pUDP2 for GT cloning |
|  | GGGGACCACTTTGTACAAGAAAGCTGGGTAGACCTAGCCGGGTCGTCATC |  |
| 5 | GGGGAGAAGTTTGTACAAAAAAGCAGGCTCCATGGCAACTATTGCATTCTC | *OsTBP* amplification from pUDP2 for GT cloning |
|  | GGGGACCACTTTGTACAAGAAAGCTGGGTATCTGAGTTCTCCACTAAATT |  |
| 6 | AATAGCTGCGCCGATGGTTTCTACA | Hygromycin amplification |
|  | AACATCGCCTCGCTCCAGTCAATG |  |

**Table S4**. Primer pairs used in qPCR analysis.

| eIF-4a (LOC_Os02g05330) | Eukaryotic initiation factor 4a | TTGTGCTGGATGAAGCTGATG | Rice reference genes |
| --- | --- | --- | --- |
|  |  | GGAAGGAGCTGGAAGATATCATAGA |  |
| 25S rRNA (LOC_Os09g01140) | 25S ribosomal RNA | AAGGCCGAAGAGGAGAAAGGT |  |
|  |  | CGTCCCTTAGGATCGGCTTAC |  |
| UBC (LOC_Os02g42314) | Ubiquitin-conjugating enzyme E2 | CCGTTTGTAGAGCCATAATTGCA |  |
|  |  | AGGTTGCCTGAGTCACAGTTAAGTG |  |
| *ACT11* (LOC_Os03g50885) | Actin 11 | CAGCCACACTGTCCCCATCTA |  |
|  |  | AGC AAGGTCGAGACGAAGGA |  |
| UBQ5 (LOC_Os01g01010) | Ubiquitin 5 | ACCACTTCGACCGCCACTACT |  |
|  |  | ACGCCTAAGCCTGCTGGTT |  |
| UBQ10 (LOC_Os02g06640) | Ubiquitin 10 | TGGTCAGTAATCAGCCAGTTTGG |  |
|  |  | GCACCACAAATACTTGACGAACAG |  |
| 18s rRNA (LOC_Os09g00999) | 18S ribosomal RNA | CTACGTCCCTGCCCTTTGTAC |  |
|  |  | ACACTTCACCGGACCATTCAA |  |
| GAPDH (LOC_Os04g40950) | Glyceraldehyde-3-phosphate  Dehydrogenase | AAGCCAGCATCCTATGATCAGATT |  |
|  |  | CGTAACCCAGAATACCCTTGAGTTT |  |
| β-TUB (LOC_Os01g59150) | Beta-tubulin | GCTGACCACACCTAGCTTTGG |  |
|  |  | AGGGAACCTTAGGCAGCATGT |  |
| eIF-1α (LOC_Os03g08020) | Eukaryotic elongation factor  1-alpha | TTTCACTCTTGGTGTGAAGCAGAT |  |
|  |  | GACTTCCTTCACGATTTCATCGTAA |  |
| F-BOX (At5g15710) | F-box protein | GGCACTCACAAACGTCTATTTC | Tobacco reference genes |
|  |  | ACCTGGGAGGCATCCTGCTTAT |  |
| L23 (At2g39460). | 60S ribosomal protein | AAGGATGCCGTGAAGAAGATGT |  |
|  |  | GCATCGTAGTCAGGAGTCAACC |  |
| OsTH1 (LOC_Os12g09000) | phosphomethylpyrimidine kinase/thiamin-phosphate pyrophosphorylase | GATGCTGCATGCTGTTCAGG | Thiamin biosynthe-tic genes |
|  |  | TGTATGGCGGACACTTGAG |  |
| OsTHI1 (LOC_Os07g34570) | Thiamin thiazole synthase | CGCTATTGTGAGGTTGACCAGA |  |
|  |  | CAAAAGTTGGTCCCATTCTCG |  |
| OsTHIC (LOC_Os03g47610) | Phosphomethylpyrimidine synthase | GAAGCATTTTGACACATACGAC |  |
|  |  | CACCTAACTTCTCCCTCCTG |  |
| EcTBP (thiB) | Thiamin-binding periplasmic protein | GCTGGTGGAGTCCGATCAAA | *E. coli* TBP |
|  |  | GAGGGGCATCGTCACCATAG |  |
| ZmTBP (GRMZM2G174883) | Legumin 1 | GCGCAACACTGGCTTTACAA | *Z. mays* TBP |
|  |  | CTCTGCAAAATGGGGTTGGC |  |
| OsTBP (LOC_Os02g14600) | Glutelin type-B 5 | CCGCTTAGGAAAGTGAGGCA | *O. sativa* TBP |
|  |  | TAGGCCAGGAGTGTTGGAGT |  |
| FeTBP (FA02) | 13S globulin seed storage protein 1 | CCGTCCTATTCTAACGCCCC | *F. esculentum* TBP |
|  |  | GTTGATCGCGTTGAGACTGC |  |
